# What Is New in the miRNA World Regarding Osteosarcoma and Chondrosarcoma?

**DOI:** 10.3390/molecules22030417

**Published:** 2017-03-07

**Authors:** Gaia Palmini, Francesca Marini, Maria Luisa Brandi

**Affiliations:** Department of Surgery and Translational Medicine, University of Florence, Florence 50134, Italy; gaia.palmini@unifi.it (G.P.); francesca.marini@unifi.it (F.M.)

**Keywords:** miRNAs, osteosarcoma, chondrosarcoma, oncogene, tumour suppressor

## Abstract

Despite the availability of multimodal and aggressive therapies, currently patients with skeletal sarcomas, including osteosarcoma and chondrosarcoma, often have a poor prognosis. In recent decades, advances in sequencing technology have revealed the presence of RNAs without coding potential known as non-coding RNAs (ncRNAs), which provides evidence that protein-coding genes account for only a small percentage of the entire genome. This has suggested the influence of ncRNAs during development, apoptosis and cell proliferation. The discovery of microRNAs (miRNAs) in 1993 underscored the importance of these molecules in pathological diseases such as cancer. Increasing interest in this field has allowed researchers to study the role of miRNAs in cancer progression. Regarding skeletal sarcomas, the research surrounding which miRNAs are involved in the tumourigenesis of osteosarcoma and chondrosarcoma has rapidly gained traction, including the identification of which miRNAs act as tumour suppressors and which act as oncogenes. In this review, we will summarize what is new regarding the roles of miRNAs in chondrosarcoma as well as the latest discoveries of identified miRNAs in osteosarcoma.

## 1. Introduction

In the last century, one of the most important scientific discoveries has been the identification of RNAs without coding potential. Scientists have reported the presence of these RNAs, which are known as non-coding RNAs (ncRNAs), based on their peculiarity. This heterogeneous group of molecules is classified based on their size as: (1) long non-coding RNAs (lncRNAs), which are longer than 200 base pairs; (2) small non-coding RNAs, which have a maximum length of 200 base pairs; or (3) circular RNAs (circRNAs), which are small covalently closed circular loop structures with either 5′ to 3′ polarity or polyadenylation at the 3′ ends [1]. Among these subclasses, microRNAs (miRNAs or miRs) are the most extensively well-known and well-studied ncRNAs. Because these molecules appear to play an important role in cellular biology, more investigations are necessary to elucidate their true functions. 

If we look at the history of this research field, which can be defined as the “world of ncRNAs”, it started in the mid- to late-twentieth century. In 1993, Lee et al. [2] discovered for the first time the presence of small RNAs during their research on *Caenorhabditis elegans*; these molecules were able to control biological processes, including the regulation of gene expression [3,4,5]. 

In their studies, Lee and colleagues observed the presence of an ncRNA and, believing that this molecule could exert temporary effects, they defined this as a small temporal RNA (stRNA) called lin-4. 

Lin-4 has been observed to regulate the transcription of proteins such as lin-14 and thus standardize the timing of larval development in *C. elegans*.

After some years, Reinhart et al. [6] discovered, while also working on *C. elegans,* another stRNA called lethal-7 (let-7). This molecule was reported to be involved in the translational repression of the gene lin-41, which participates in the larval development of *C. elegans*. 

After the discovery of more stRNAs in *C. elegans*, other reports began describing these same molecules in mammals [7] and humans [8]. This discovery was critical because it served as proof that these small RNAs are well conserved and acted as important gene regulators [9,10]. For this reason, this class of molecules was redefined as small non-coding RNAs (sncRNAs), which are not temporary molecules. From these first studies, scientific interest around these small molecules has increased, and the subclass of microRNAs (miRNAs or miRs) has become the most investigated and is currently the most well-known of the ncRNAs [11]. At the same time, the other two classes of ncRNAs (lncRNAs and circRNAs) have begun to garner increasing interest. 

The recent discovery of circRNAs has changed the idea about RNAs as a solely linear entity. These small well-conserved circular molecules have several properties thanks to their unique features. The first reported circRNA, Sry, acts as a sponge for miR-138 [12]. Additional studies on circRNAs are needed to understand their potential role and elucidate their effect in contributing to the diversity and complexity of eukaryotic transcriptomes.

lncRNAs include molecules that are longer than 200 base pairs; however, they are not always as well conserved as miRNAs and circRNAs [13]. Despite this, some lncRNAs are well characterized and highly conserved [i.e., X inactive specific transcript (Xist), hi antisense Tsix (TSIX transcript, XIST antisense RNA) [14] and HOX antisense intergenic RNA (HOTAIR) [15]]. In contrast, a high number of conserved lncRNA promoter sequences has been observed, which demonstrates that their regulation is very important [16]. Despite their expression levels, which are lower than those of miRNAs, lncRNAs can be found in many tissues, and they are often tissue-specific. 

lncRNAs are present in the cytoplasm and the nucleus, where the majority remain after synthesis. Their synthesis is mediated by the transcriptional machinery of RNA Polymerase II (Pol II) and by RNA Polymerase III (Pol III). Pol II transcribes lncRNAs that are associated with chromatin and are processed as classical mRNAs with transcriptional elongation and polyadenylation. However, Pol III transcribes other nonpolyadenylated lncRNAs that fold into tertiary structures [16,17,18,19]. 

Based on several studies on the localization of lncRNAs inside the genome they have been divided into intergenic, intronic, bidirectional, sense and antisense lncRNAs [20,21,22,23,24]. Because of their length, lncRNAs can form complex structures, including helices and hairpin loops, by which they can interact with DNA, RNA and proteins [13,25]. 

Recent studies have shown that lncRNAs can behave as signals or decoys by negatively regulating transcription, as guides for ribonucleoprotein (RNP) binding, as an interactive molecule that binds to target genes either in cis or in trans, and as scaffolding molecules. Regarding scaffolding activity, lncRNAs act as a platform and can create RNP complexes known as lncRNA-RNPs, which can remodel chromatin via histone methylation with the help of polycomb group proteins (PcG) [26].

Some studies have also revealed the ability of lncRNAs to not only act as transcriptional or post-transcriptional regulators but also function in a manner analogous to classical miRNAs by coding for micropeptides and engaging with ribosomes [27]. 

Another important function of lncRNAs is to regulate miRNA activity by acting as either a competitive endogenous RNA (ceRNA) or as an miRNA sponge [28,29,30]. ceRNAs have been described as lncRNAs that, having one or more sequences similar to RNAs, regulate transcription by sequestering molecules that act as regulatory transcription factors, catalytic proteins and miRNAs [30]. However, lncRNA sponges are complex structures that present binding sites for one or more miRNAs and thereby act to regulate the effects of miRNAs [31]. 

The literature on lncRNAs and miRNAs has highlighted both classes of molecules as important regulators of physiological cellular processes such as development, proliferation, apoptosis and migration [3,11,32,33]. These ncRNAs are also involved in pathological processes associated with human diseases, including cancer [34,35,36]. 

Some physiological processes that lncRNAs control include pluripotency and lineage commitment, as described for the lncRNA Tcl 1 upstream neuron-associated (TUNA) lincRNA, which are also known as TUNA factors. lincRNA can bind Sox2, Nanog and Fgf4 (three pluripotency transcription factors), thereby preventing the suppression of miRNAs and maintaining the self-renewing potential of human embryonic stem cells [37]. Another important physiological lncRNA is Braveheart (Bvht), which was discovered in a study on cardiovascular development and is fundamental in the development of cardiovascular progenitor cells [38]. A recent study showed that lncRNAs are involved in human pregnancy. In fact, the lncRNA Meg3 has been reported to be important in reducing the risk of apoptosis and promoting the migration of trophoblasts [33]. Regarding their role in pathological cellular processes, lncRNAs are involved in cardiovascular diseases [39], fibrotic diseases [40,41] and cancer development. Scientists have observed that lncRNAs can either promote or suppress several types of cancers, and an especially strong interaction between lncRNAs and miRNAs could be the basis of tumour development [16,42,43]. 

Several recent studies on the role of lncRNAs in cancer have shed light on which ones are onco-lncRNAs (enhancing tumour growth) and which ones are tumour suppressor lncRNAs (inhibiting tumour progression). A study on hepatocellular carcinoma reported that the lncRNA PTENP1 and its target tumour suppressor gene PTEN are important in mitigating tumour growth. It has been documented that both genes are lost during the progression of several cancers. Restoring the levels of lncPTENP1 leads to inhibition of tumour growth and enhanced autophagy. Further investigations revealed that lncPTENP1 acts as a sponge for miR-17, miR-19b and miR-20a, all of which target and block the expression of genes responsible for autophagy. Hence, the tumour suppressor function of lncPTENP1 is blocking these miRNAs to remove their inhibition on autophagy [44]. In contrast, lncATB has been recognized as an onco-lncRNA that is overexpressed in all breast cancer patients who exhibit resistance to trastuzumab chemotherapy. Studying the activity of lncATB, scientists observed that this lncRNA binds miR-200c, a tumour suppressor that prevents the epithelial to mesenchymal transition (EMT). Therefore, lncATB, by blocking miR-200c, promotes invasion and metastasis of cancer cells [45]. There are also recent studies demonstrating that lncRNAs can be targets for miRNAs. A report on prostate cancer suggested that the interaction between miR-34a and HOTAIR, a well-conserved lncRNA, can mitigate the oncogenic effect of HOTAIR. Furthermore, miR-34a binding to HOTAIR results in knockdown of HOTAIR, thus causing a decrease in prostate cancer cell growth and metastasis [46]. 

Another important pathological process that involves ncRNAs is tumour angiogenesis, which is fundamental for cancer development as a response to hypoxic conditions within the tumour microenvironment and as a method to supply the nutrients necessary for continued growth of the tumour bulk. Tumour angiogenesis is a complex process that results from the balance between pro-angiogenic and anti-angiogenic factors within the tumour microenvironment. Recent studies have shown how angiogenesis is modulated by ncRNAs; specifically, lncRNAs and miRNAs [47]. As in cancer development, ncRNAs they can affect each other in several ways within the process of tumour angiogenesis. One example is the interaction between miR-9 and lncMALAT1. lncMALAT1 has been described as a pro-angiogenic factor in cancer stem cells (CSCs) by acting as a sponge for miR-200c and miR-145, leading to subsequent upregulation of Sox2 and an eventual shift of the cell towards more stem-like properties. The activity of lncMALAT1 is blocked by miR-9, and activation of AGO2 by miR-9 results in the degradation of MALAT1. Thus, miR-9 inhibits the angiogenesis [48].

In summary, the last two decades have shown an increasing focus on the study of ncRNAs with respect to the physiological and pathological processes. The discovery of the molecules involved in the crosstalk among miRNAs, lncRNAs and circRNAs now represents an important challenge for scientists in elucidating the complexity of these processes. Obviously, one of the most characterized pathological processes is cancer. Although the study of lncRNAs and circRNAs in the context of cancer development and progression is in its infancy (particularly for circRNAs), there is increasing literature regarding the role of miRNAs in cancer progression. 

In fact, after identifying the importance of miRNAs in biological processes [4,49], researchers have focused more on the role miRNAs in cancer progression. [50,51] Moreover, these small non-coding RNAs, which play an important and complex role in post-transcriptional gene expression, can be used as prognostic, diagnostic and predictive markers, but they can also be used as targets in the development of new therapies [52,53]. 

Targeting miRNAs could be a valid solution for the challenges posed by researchers regarding the possibility of developing novel and more effective anti-cancer therapies. In particular, for tumours that are rare, have unknown aetiology and, despite the existence of aggressive therapies, have a poor prognosis, there is often a lack of effective therapy. 

One example of these difficult-to-treat tumours is sarcomas, specifically, bone sarcomas. Despite available multimodal treatments, which include chemotherapy, surgery and radiotherapy, the prognosis of these patients is still often poor. There are three types of primary bone sarcomas: Ewing’s sarcoma, chondrosarcoma and osteosarcoma. These are very rare tumours characterized by a poor 5-year survival rate from the time of diagnosis, despite recent advances treatment [54] Hence, the scientific world has started to consider the role of miRNAs in these sarcomas in an attempt to elucidate the tumour aetiology. The ultimate goal is to identify new molecular targets and develop more effective anti-cancer therapies. 

In this review, we provide a detailed description about the current progress of miRNA research regarding osteosarcoma (OS) and chondrosarcoma (COS). We first provide an overview on the role of miRNAs in physiological osteogenesis after a focused analysis on which roles are the bone cancer-associated roles of miRNAs. 

## 2. Biogenesis and Functions of miRNAs

After the discovery of these small RNA molecules in *C. elegans* (which have the ability to regulate several biological processes) [2], the subsequent search for endogenous RNAs that can silence genes has resulted in the discovery of a large family of small RNAs. The major class of this family is known as microRNAs (miRNAs or miRs) [8,55,56]. 

Aside from miRNAs, two other classes of small RNAs have been identified—endogenous small interfering RNAs (siRNAs) and Piwi-interacting RNAs—both of which are important in genetic regulation [57,58]. miRNAs are endogenous single-strand RNAs 18-24 nucleotides in length with the ability to regulate eukaryotic gene expression at the post-transcriptional level [59]. In 2003, over 200 miRNAs were experimentally discovered, and several have been identified using computational approaches. Currently, over 2500 human miRNAs have been identified, and it is highly likely that two-thirds of all human genes are directly targeted by these miRNAs [60,61]. However, what is the origin of these endogenous small RNAs?

There are three different miRNA biosynthesis pathways. Two of these pathways are considered alternative processing pathways; one regulates the biogenesis of only a small subset of miRNAs known as mirtrons [62], and the other is related to the biogenesis of miR-451 [63]. The third pathway is the canonical miRNA synthesis pathway.

Biogenesis starts in the nucleus where the miRNA genes are transcribed by RNA Polymerase II to form primary hairpin transcripts (pri-miRNAs); afterwards, the pri-miRNAs are processed into precursor miRNAs (pre-miRNAs) by an RNase endonuclease III called DROSHA, which is always localized inside the nucleus. 

The pre-miRNAs, which have a stem-loop secondary structure, must be exported to the cytoplasm via the nuclear protein Exportin-5, a Ran-GTP-dependent transporter. Upon translocation into the cytoplasm, the pre-miRNAs are processed by a second RNase III enzyme known as DICER. 

At this point, the pre-miRNAs are now double-stranded miRNAs, which can form an active RNA-induced silencing complex (RISC). This complex comprises a double-stranded miRNA associated with the Argonaute 2 protein (AGO2), the key catalytic enzyme within the complex. Thus, RISC is the necessary complex by which miRNAs post-transcriptionally silence gene expression. 

In this complex, mature miRNAs usually bind their target mRNAs at the 3′ untranslated region (3′ UTR) at a sequence of 6-8 base pairs called the “seed region” at the 5′ end of the mature miRNAs. The short miRNA-mRNA binding site permits each miRNA to target more than one mRNA [62,64,65]. This described system is highly regulated and controls expression at the transcriptional level. This allows for the tissue- and cell type-specific expression of miRNAs. The regulatory mechanisms act on the two enzymes DROSHA and DICER, which are regulated by Di George syndrome critical region 8 (DGCR 8) and TAR RNA-binding protein (TRBP), respectively [66,67]. Several studies on miRNAs have shown that one miRNA, based on its seed region, can target ~30% of expressed genes and is highly tissue-specific and well conserved [68]. 

miRNAs are implicated in several physiological processes, such as development, proliferation, differentiation and apoptosis in normal cells [4,69]. They are also involved in the maintenance of the pluripotency. Different studies have showed the role of some miRNAs (i.e., miR-15a, miR-16, miR-19b, miR-302 cluster, miR-290, let-7 miRNAs a, miR-34a and miR-145) in the physiology of embryonic stem cells (ESCs) [18,70,71,72,73]. As several studies have suggested, miRNAs can either re-enforce the pluripotent state or promote differentiation such that the final outcome in the cellular system is synergistic and balanced. 

Given these observations that the miRNAs mentioned above can act as promoters or suppressors of pluripotency in ESCs, Zovoillis et al. [74] studied how overexpression of the miR-290 family enhances cell proliferation by targeting p21, an inhibitor of cell proliferation, and maintains pluripotency because it safeguards the expression of Lin28, an RNA-binding protein that prevents the maturation of the miR let-7a, a strong promoter of cell differentiation and an inhibitor of miR-290 [75].

The realization that miRNAs are key molecules in normal cellular processes has paved the way for the investigation of their roles in pathological conditions, especially cancer. Since their discovery, the influence of miRNAs has been observed at several levels in the pathogenesis, progression, development and invasion of cancer [53]. 

## 3. The Many Roles of miRNAs in Cancer: Oncogenes, Tumour Suppressors, Biomarkers and Therapeutic Targets

In 2002, Calin et al. described for the first time that miRNA dysregulation could translate into the manifestation of chronic lymphocytic leukaemia [50]. After their discovery, researchers began to analyse how miRNAs contribute to diseases and disorders such as cardiovascular diseases [76], fibrosis [77], diabetes [78], neurodegenerative diseases [79] and cancer [80]. In relation to their expression and function inside the tumour bulk, miRNAs can behave either as an oncogene (onco-miR) or as a tumour suppressor, and their function also changes in relation to the role of their mRNA targets [80]. Based on the study regarding the functions of miRNAs in disease, scientists have realized that these small molecules can be not only targets for the development of new therapies but also be biomarkers for the same diseases. Obtaining a correct diagnosis early in the disease progression is an important aspect for successfully treating diseases, especially tumours. A correct and prompt diagnosis is fundamental fora good prognosis and a good response to anti-cancer treatments [53]. To date, the diagnostic standard of cancer diagnosis is often the histopathological analysis of a tumour sample, which is usually obtained from a surgical biopsy. This method is not only invasive and expensive but can also be dangerous for patients. So, finding new methods could be useful to improve the diagnosis. One solution is identifying molecules that are specifically related to diseases and can be detected within samples of biological fluids from humans. Consequently, since the discovery of extracellular and circulating miRNAs, a probably approach appears to involved miRNAs. In 1947, Mandel and Metais [81] observed the presence of circulating RNA and DNA in the plasma of healthy and sick individuals. In the 1960s, circulating free nucleic acids (cfDNA/RNA) were detected in patients with autoimmune diseases [82]. In 1977, Leon et al. [83] postulated for the first time the potential of cDNAs as biomarkers. In 2007, scientists discovered the presence of miRNAs in the blood of a lymphoma patient [84]. 

Corsten et al. observed an increase in the levels of miR-208b and miR-499 in blood from patients who recently experienced a myocardial infarction [85]. They hypothesized that this phenomenon was related to a passive release of miRNAs into bodily fluids after tissue injury, cellular apoptosis or necrosis. After these first studies on the presence of miRNAs in the body fluids (i.e., blood, saliva, and urine) of both healthy and diseased people, the field of circulating miRNAs has expanded. Several studies have shown that these molecules are highly stable in bodily fluids and are packaged into exosomes [86,87], which are small (40–100 nm) membraned vesicles of endocytic origins that are released by cells into the extracellular environment. These microvesicles might be a communication method among cells. Exosomes have been studied in several cellular processes such as antigen presentation on T-cells and metastasis [88]. 

Several cells are capable of releasing exosomes, and the composition (lipids, proteins) of the vesicular membranes is an indicator of the origin and function of exosomes. Valadi et al. first revealed the presence of small ncRNAs inside exosomes from cells [89], and Hunter and his collaborators subsequently reported the presence of miRNAs in circulating plasma exosomes from healthy patients [90]. In relation to this, scientists have started to think that individuals can be identified using miRNAs circulating exosomes as a fingerprint; this method could distinguish healthy and pathological conditions and serve as a novel diagnostic, prognostic and disease surveillance tool. Unfortunately, we have yet to specifically and clearly identify all the miRNAs that are associated with pathological disorders and cancer subtypes. Several research groups are working hard to identify which miRNAs are specific for each different cancer type, which ones are always present in the bodily fluids of patients, and which ones are possibly modulated in response to anti-cancer therapies. Additionally, the origin of these exosomes containing miRNAs is also unclear. They can be released by necrotic tumour cells, lysed tumour cells, cells from another tissue affected by an ongoing disease, or the tumour itself, which actively secretes miRNAs into the surrounding environment. Therefore, further studies in this field are necessary. However, although we do not have the entire map of all the miRNAs that are specifically involved in a disease, we have identified some miRNAs that may be specific to a disease and its stage; thus, these can be used as biomarkers [91]. 

In recent years, many studies have been published on how to detect miRNAs within bodily fluids with the goal of applying the detection of miRNAs as a clinical diagnostic tool [92]. Several detection methods for miRNA analysis have been implemented in a clinical setting [93]. The gold standard is represented by amplification-based methods [94,95,96], followed by hybridization-based methods [97,98] and microarrays [99], which are another powerful method that can also detect unknown miRNAs. More recent techniques are based on capillary electrophoresis-mass spectrometry [100]; the creation of new and simpler microarray platform that does not require RNA extraction, labelling and target amplification [101] and a technique based on the use of a label-free miRNA electrochemical biosensor [102]. In relation to this, before translating advanced technologies like miRNA serum profiling into clinical practice, certain problems should be addressed. One is that the miRNA analysis methods should be standardized; to achieve this, it is necessary to use the same extraction methods, standardized platforms, target miRNAs and body fluids. However, we are still far from this goal, and more research must be performed to develop a completely standardized protocol for the detection of miRNAs in clinical practice.

Another important aspect critical for the future application of miRNA detection in clinical practice is determining which miRNAs are specific for each cancer type and understanding the role of these miRNAs in cancer progression (i.e., whether they are tumour suppressors or promoters) [92,93,103,104]. Recent studies have shown that miRNAs can act as tumour suppressors by targeting specific genes or lncRNAs, which are known to be oncogenes, and subsequently blocking their cancerous potential by inhibiting cancer invasion and proliferation. For example, miR-1, which has been described as an important factor in the development of skeletal and myocardial muscle, acts as a tumour suppressor in several cancers (i.e., lung cancer, hepatocellular carcinoma, colon cancer, OS, etc.); in fact, miR-1 is downregulated in all these malignancies. 

This is related to the ability of miR-1 to target several genes that encode for proteins involved in cancer development and proliferation [105,106]. miR-1 can also target the lncRNA UCA1, thus inhibiting its expression and subsequently increasing cell apoptosis and motility [107]. 

Members of the let-7 family are tumour suppressor miRNAs that inhibit the MAPK/ERK pathway [108]. On the contrary, there are miRNAs that enhance cancer development and progression. 

A recent study on miR-10b showed that this miRNA is overexpressed in several types of cancer, including oesophageal cancer, breast cancer and glioma; in these systems, miR-10b acts to promote cell mobility and invasiveness by targeting an important gene involved in these processes and enhancing its activity [109]. Other similar examples are miR-21, miR-155 and miR-29, which are, in fact, overexpressed in the respectively cancerous tissue and thus are also related to the poor prognosis observed in these patients [110,111,112]. In the last century, evidence regarding the different roles that miRNAs play in physiological and pathological conditions such as cancer has paved the way for the development of new therapeutic molecules, which can directly act on individually target miRNAs and enhance their onco-suppressor potential. For example, the first miR-based clinical trial used an miR-34 mimetic (MRX34, Mirna Therapeutics, Austin, TX, USA) to restore the levels of the tumour suppressor miR-34 in several types of cancer in which miR-34 has been downregulated [113]. 

## 4. miRNAs in Bone

miRNAs are a class of small non-coding RNAs that can regulate gene expression at the transcriptional and post-transcriptional level simply by binding to specific sequences present on the target genes. As mentioned above, miRNAs are important regulators of not only pathological conditions but also physiological processes, including bone development. Osteogenesis is a complex process characterized by the balance between osteoclastogenesis and osteoblastogenesis. These activities are controlled by an elaborate signalling network, which includes growth factors, transcription factors, effectors and miRNAs. Several studies on the role of miRNAs in the three principle processes involved in the construction of the human skeleton (i.e., osteoclastogenesis, osteoblastogenesis and chondrogenesis) have elucidated the contributions of miRNAs regarding bone development. Of the miRNAs discovered, 22 have been identified as inhibitors of the BMP-2-induced osteoblast differentiation pathway [114]. miR-378, miR-322 and miR-196a have been identified as involved in BMP signalling by promoting BMP-induced osteogenic differentiation. Other pathways such as Wnt/β-catenin, Notch and TNF-α signalling are involved in osteoblastogenesis. Many studies have demonstrated that all these signalling pathways are regulated by different miRNAs, which can promote the osteogenic differentiation of mesenchymal stem cells (MSCs) [115]. For example, miR-346 promotes the osteogenic differentiation of MSCs by targeting the glycogen synthase kinase-3β (GSK-3β), which is important in the activation of β-catenin—a critical factor in the Wnt/β-catenin pathway [116]. There are reports indicating that high levels of miR-34a inhibit the Notch signalling pathway resulting in the expression of RUNX2, OSX and OCN, which are fundamental genes in osteogenesis [117]. Meanwhile, miR-21 promotes the osteogenic differentiation of MSCs by inhibiting the TNF-α signalling pathway [118]; however, several other miRNAs suppress the osteogenesis of MSCs. For example, miR-206 levels decrease during osteogenesis differentiation. In fact, it has been demonstrated that overexpression of miR-206 inhibits osteoblast differentiation, whereas knocking out miR-206 enhances this process [119]. miR-26a and miR-196a have been described as inhibitors of osteogenic differentiation of adipose-derived stromal cells [120,121]. 

There are also miRNA promoters and inhibitors that regulate osteoclastogenesis [122]. Studies focusing on the promoters have shown that members of the miR-29 family are overexpressed during osteoclastogenesis to form bone marrow monocytes. This overexpression has also resulted in an increase of the osteoclast markers TRAP and cathepsin K [123]. In contrast, knockdown of miR-29 inhibited the commitment and migration of pre-osteoclasts, thereby blocking the osteoclastogenesis process [124]. 

miR-223-3p is another well studied miRNA in osteoclastogenesis; downregulation of this miRNA is characteristic of osteoclast differentiation. Although overexpression of miR-223-3p blocks osteoclast differentiation, inhibition of this miRNA results in the formation of TRAP-positive osteoclasts [125]. This suggests that maintaining the correct levels of miR-223-3p is necessary to promote appropriate osteoclast formation. Finally, chondrogenesis is a fundamental process for the development of a healthy skeleton. Recent studies have observed that miR-140 negatively targets histone deacetylase 4 (HDAC4), particularly expressed in non-hypertrophic chondrocytes. HDAC4 regulate chondrocyte hypertrophy by inhibiting Runx2. When miR-140 binds to HDAC4, the inhibition on Runx2 is removed, and chondrocyte hypertrophy, a process that is fundamental for proper endochondral ossification, is enhanced [126]. In contrast, miR-145 targets Sox9 gene to inhibit the expression of the cartilage genes while concurrently promoting Runx2 expression [108]. Even though several miRNAs have been reported to be involved in normal bone development, many other individual miRNAs could contribute to this activity, and their roles have not yet been discovered; therefore, further studies in this vein are necessary. Regarding the observations that miRNAs are involved in human diseases and that the miRNA dysregulation could contribute to the development of cancer, it has become increasingly clear to scientists that the osteogenesis process could also be influenced by miRNA levels. Recent studies have shown that primary bone tumours, including Ewing’s sarcoma, chondrosarcoma, OS and giant cell tumours, change their miRNA expression pattern during their progression [127]. Obviously, extensive and complete knowledge of the target miRNAs specific to each type of cancer should be collected for the future development of anti-cancer therapy as well as for the establishment of new diagnostic methods based on the detection of circulating miRNAs.

## 5. miRNAs and Primary Bone Tumours

### 5.1. miRNAs in Chondrosarcoma

As we have previously reported, miRNA expression can be altered in malignancies and play a fundamental role in tumour progression. This is also the case for primary bone tumours. In fact, altered miRNA expression has been reported in human chondrosarcoma (COS). 

COS, another primary skeletal sarcoma, is characterized by the abnormal production of a specific cartilage matrix. Although COS can manifest in any bone, this cancer preferentially develops in the pelvis, humerus, femur, scapula and ribs and can present at any age [128]. Based on the histological profile, four different subtypes of COS have been identified: (a) conventional COS, which is the most common (approximately 90% of diagnosed cases); (b) dedifferentiated COS (approximately 10% of diagnosed cases); (c) mesenchymal COS, the most rare but also the most aggressive; and (d) clear cell chondrosarcoma, a rare variant. COS has been shown to be resistant to the chemotherapy and radiotherapy. Consequently, an improved prognosis for patients is based on an extremely precise surgery, but unfortunately, patients with COS usually relapse and inevitably have a poor prognosis [129]. Currently, the discovery of new molecular diagnostic and prognostic biomarkers represents the only approach to develop effective and specific treatments against COS. 

**Figure 1:**
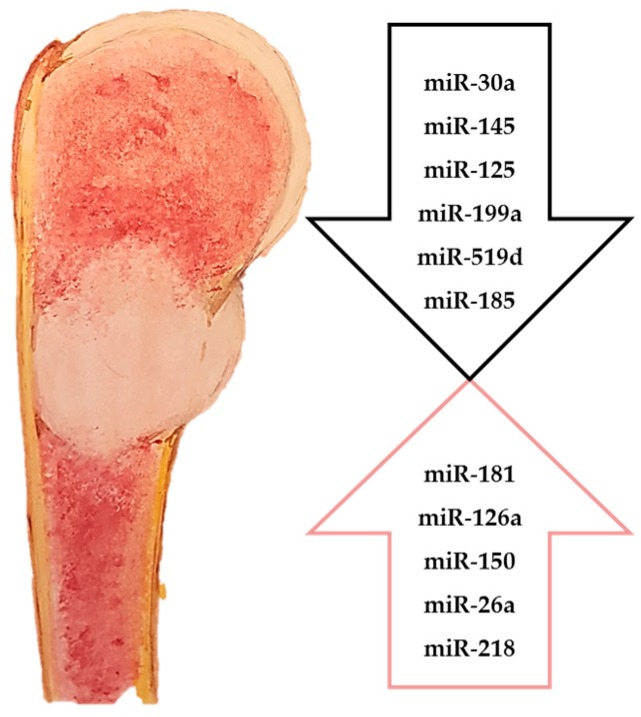
Downregulated and upregulated miRNAs in chondrosarcoma.

Therefore, miRNAs could serve as these biomarkers as well as possible novel targets for the development of future therapies. Several studies have been conducted regarding the roles of miRNAs in COS progression (Figure 1).

It has been observed that miR-30a exerts two different functions in COS. Lu et al. reported that miR-30a could decrease tumour proliferation, migration and invasion using a miR-30a mimic on a human chondrosarcoma cell line [130]. They also observed that miR-30a expression was negatively correlated to the tumour grade. In fact, in advanced stage COS, the miR-30a levels were very low. Furthermore, they reported that one target of miR-30a is SOX4, and this interaction subsequently reduced cancer cell growth and invasion. SOX4 is a member of the SRY-related HMG box (SOX) gene family, which is implicated in chondrogenesis [131]. Therefore, SOX4 is a key gene in COS progression. Interestingly, Lu et al. reported that in cancerous tissues with lower levels of miR-30a, SOX4 is overexpressed. In summary, this study suggested that SOX4 is an oncogene and is regulated by miR-30a in COS cells, thus highlighting the important role of miR-30a as a tumour suppressor [130]. Additionally, Jiang et al. reported that downregulation of miR-30a enhances cancer progression. miR-30a negatively targets RUNX2 in COS cells, and inhibits to this gene to enhance the cancer invasion [132]. SOX9, another member of the SOX family, is overexpressed in COS and has been implicated in cancer progression [133]. A recent study showed that downregulation of SOX9 increases the apoptosis rate [134]. One of the target genes of SOX9 is ETV5 [135], an effector of EMT and a metastatic target in cancer. Mak et al. investigated the regulation of these two genes and discovered that miR-145, which has reduced expression in COS, targets and inhibits SOX9. The inhibition of SOX9 is responsible for the downstream activation of ETV5, which then activates MMP-2 and results in the induction of invasion. Therefore, miR-145 suppresses COS metastasis [136].

Regarding the role of miRNAs in response to chemotherapy and radiotherapy, Tang et al. demonstrated in a study using different COS cell lines that miR-125b could act as a tumour suppressor by inhibiting glucose metabolism and targeting the oncogene ErbB2. They also showed that when miR-125b was downregulated, all the COS cell lines developed resistance to doxorubicin, a chemotherapy agent. In contrast, overexpression of miR-125b enhanced the cells’ sensitivity to this drug [137]. 

Another aspect that has been investigated in COS is cancer cell response to hypoxic conditions. Hypoxia, which has been reported to be the source of dysregulated gene expression in cancer, is likely responsible for some cancer traits, one of which is cancer-induced angiogenesis. As described above, angiogenesis is fundamental for tumour progression and provides cancer cells a means by which to access the nutrients essential for their proliferation. 

Angiogenesis is a complex process characterized by the interplay of pro-angiogenic (e.g., VEGF/VEGFR and PDGF/PDGFR) and anti-angiogenic factors (e.g., TSP-1/TSP-2). An in vitro study on COS cells grown in hypoxic conditions has shown that miR-181a is tightly correlated to the promotion of angiogenesis [138]. It has been demonstrated that miR-181a (once activated) can directly target the VEGF gene and enhance its expression, thereby inducing angiogenesis. In a study of miR-181a function in COS, researchers reported that miRNAs can function as an oncogene. Furthermore, miR-126 [139], miR-150 [140], and miR-26a [141] have been shown to play a role in promoting angiogenesis. 

Liu et al. also studied the angiogenesis process in COS. By focusing on the role of the chemokine CCL5 (which has been reported to be related to cancer migration and metastasis in other cancers as well as), they reported that CCL5 induces VEGF upregulation. This occurs with concomitant downregulation of miR-199a. In fact, Liu and colleagues demonstrated that co-transfection of COS cells with a miR-199a mimic completely abolishes CCL5-mediated VEGF expression and angiogenesis [142]. Other recent studies on miRNAs in COS showed that miR-519d, miR-185, and miR-218 play important roles in COS progression.

miR-519d has been correlated to the invasive capacity of COS because of its ability to interact with matrix metalloproteinases (MMPs), which are the key molecules that promote tumour metastasis. Several studies have reported that among the MMPs, MMP-2 is the most critical in COS because it can degrade type IV collagen, the primary component of cartilage [143]. In their study, Tsai et al. demonstrated that the migratory potential of COS is elevated in cases with MMP-2 overexpression and also observed that the concomitant levels of miR-519d were very low. In fact, when the miR-519d levels are restored, a decrease in the MMP-2 levels and inhibition of the migratory capacity were observed [144]. Another study conducted by Goudarzi et al. on the role of miR-185 and miR-218 expression in patients with COS showed that (using real-time PCR analysis) patients with a profile characterized by the contemporary upregulation of miR-218 and the downregulation of miR-185 have a poor survival rate [145]. As indicated by researchers, the observed low survival rate should be correlated to the decrease of miR-185 levels during COS progression as has been reported in other cancers [146,147]. In fact, several studies on other tumours have documented that miR-185 overexpression is the basis of inhibiting cancer cell proliferation. At the same time, it has been observed that while miR-185 is downregulated during cancer progression, miR-218 is overexpressed [148]. In conclusion, Goudarzi et al. demonstrated the specific activities of both these miRNAs in the enhancing the COS development. miR-218 acts as an oncogene to support COS progression, whereas activated miR-185 functions like a tumour suppressor [149]. All the discoveries here are illustrated and summarized in Table 1. Regarding the role of miRNAs in the biological processes of COS, the available literature provides an initial understanding of their roles in influencing the proliferation, migration, invasion and chemoresistance of COS. In addition, it encourages further studies to better elucidate their functions and discover other miRNAs involved in COS. The culmination of this field aims to identify unique and valid targets for the future development of novel therapies against COS.

### 5.2. miRNAs in Osteosarcoma

**Figure 2:**
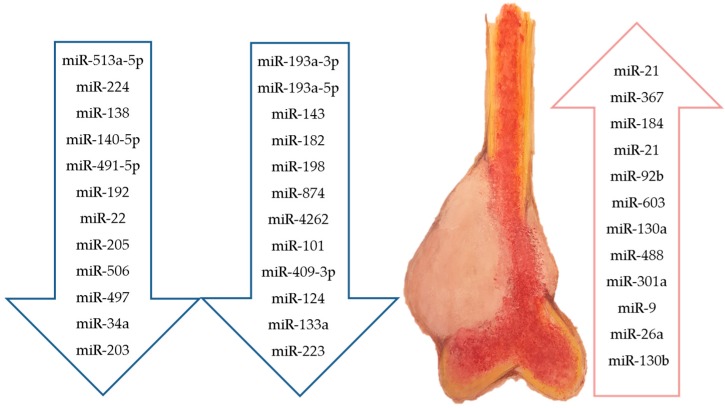
Downregulated and upregulated miRNAs in osteosarcoma.

OS, another primary bone tumour, often occurs in children and adolescents [150]. Based on the histological analysis, it has been observed that several subtypes of OS (including COS) exist. Among them, the most commonly diagnosed is conventional OS, which can be osteoblastic, chondroblastic or fibroblastic depending on the histology. OS principally arises from the metaphysis regions of the long bones where there is active bone remodelling, suggesting that the initial onset of this cancer is likely because of dysregulated or inappropriate signalling during remodelling. Currently, despite the availability of multimodal therapy (i.e., surgical resection of the tumour bulk combined with chemotherapy and/or radiotherapy), the 5-year survival rate of OS remains lower than 70% [151]. This poor survival rate is why OS is currently the second leading cause of cancer-related death in children. Obviously, the recent discovery of miRNAs has been studied as a possible solution to better understanding the molecular basis of the biology of this aggressive bone cancer. Consequently, many studies have been conducted regarding the association of miRNAs with human cancers, including OS, [127]. In relation to this, we have decided to summarize all the research that has been conducted in the last year regarding the expression of miRNAs (Figure 2) and the two principle roles of miRNAs in the progression of OS. These two areas are their role in the acquired resistance to multidisciplinary treatments and how they contribute to cancer development and progression. 

#### 5.2.1. miRNAs as New Players in Osteosarcoma Chemosensitivity and Chemoresistance: The Latest Discoveries

An important aspect in which miRNAs have been implicated in cancer is the development of chemosensitivity and chemoresistance [152], with OS [153] presenting some of the most aggressive characteristics and most resistant responses to therapies. One component of the multidisciplinary therapeutic approach against OS is radiotherapy, which is usually performed after surgery to ensure elimination of the entire tumour bulk. Unfortunately, several cases of radioresistance have been reported [154]. Consequently, it became clear that to understand the cellular mechanisms of chemotherapy and radiotherapy resistance, it will be useful to identify molecules that are specific to OS not only to improve the knowledge base of OS biology but also to develop therapies to target these molecules; this approach could be more efficient and less invasive. In relation to this, we have identified many studies published within the last year that focused on which miRNAs exert functions in OS.

In their in vivo and in vitro studies, Dai et al. detected the presence of the human apurinic/apyrimidinic endonuclease/redox effector factor (APE1) in OS cells; this protein is responsible for repairing DNA damage caused by ionizing radiation and the subsequent presence of reactive oxygen species. In fact, overexpression of APE1 has been reported in several cancers [155,156,157]. Dai and colleagues also demonstrated not only overexpression of APE1 but also simultaneous downregulation of miR-513a-5p. Interestingly, the levels of miR-513a-5p were higher when APE1 is knocked down; therefore, there exists an inverse relationship between miR-513a-5p and APE1 expression. Furthermore, they demonstrated that restoring the levels of miR-513a-5p can sensitize the cells to ionizing radiation. This negatively targets APE1, and decreased expression of this gene enhances the radiosensitivity and induces subsequent apoptosis [158].

The other therapeutic treatment targeting OS that is combined with surgery and radiotherapy is adjuvant chemotherapy, which is based on the use of cisplatin (Cis), doxorubicin (Dox), methotrexate (Met) and adriamycin (Adr). Similar to radiotherapy, many OS patients begin to demonstrate chemoresistance to chemotherapy. Therefore, scientists have started to investigate the reason for this phenomenon and studied the role of miRNAs on the sensitivity of cancers to chemotherapeutic agents. Within the last year, miR-21, miR-224 and miR-138 appeared on the oncology research scene as primary players in the cancer response to chemotherapy [159,160,161].

miR-21 was shown to be upregulated in several tumours (i.e., colorectal, lung, breast and liver) as well as in OS [162,163,164,165]. miR-21 targets several genes that act for the most part as tumour suppressors which inhibit cell apoptosis, cell signalling, cell proliferation and migration.

Vanas et al. observed that a reduction of miR-21 activity leads to an inhibition of cell proliferation and migration in OS cell lines, whereas upregulation of miR-21 accelerates the doubling rate of these cells. Furthermore, they also demonstrated that miR-21 expression confers chemoresistance to OS cells by decreasing the cellular sensitivity towards Cis by targeting Spry1 and Spry2 genes [159]. However, this activity has no effect on the sensitivity to either doxorubicin or methotrexate. This aspect has been observed also in other tumours [166,167]. Geng et al. demonstrated that low levels of miR-224 in OS cell lines and tissues should be correlated with a shorter survival outcome. In their study, they demonstrated that overexpression of miR-224 in OS cell lines could inhibit proliferation, migration and invasion and as well as increase the sensitivity to Cis. All these effects are controlled by the gene Rac1, which is a direct target of miR-224. The same study also reported that Rac1 is upregulated in OS cell lines and tissues and is responsible for the tumour progression and the development of chemoresistance to Cis. Interestingly, Rac1 downregulates miR-224. Therefore, Geng and colleagues showed that there is an inverse correlation between Rac1 and miR-224 and that miR-224 overexpression in OS cells represses Rac1 and enhances the chemosensitivity to Cis [161]. Obviously, the repression of miR-224 by Rac1 is an indicator of possible chemoresistance, and, accordingly, a poor survival rate. The more recent miRNA that was shown to be involved in Cis resistance is miR-138, which has been observed to have reduced expression in OS tissue than in normal tissue. This decrease might be related to its tumour suppressor capacity. As scientists have observed, if the levels of miR-138 are restored, there is a dramatic inhibition of cell proliferation and invasion as well as an increase of chemosensitivity to Cis. They also demonstrated that the change in chemosensitivity can be partially abolished by overexpression of the EZH2 gene, which can block the activity of caspase-3, a critical enzyme for apoptosis. 

Therefore, this study revealed that in OS, EZH2 is the specific target gene for miR-138 and that this miRNA acts as a tumour suppressor in OS by enhancing the chemosensitivity to Cis [161]. In contrast, Wang et al. demonstrated for the first time that miR-367 plays a key role in the development of chemoresistance in OS [168]. Recent studies revealed that miR-367 executes several functions in tumours [169,170,171] and acts as an onco-miRNA in OS. In this study, they observed that the OS cell lines that overexpress miR-367 exhibit strong resistance to treatment with adriamycin (Adr). This effect is mediated by the decrease of the expression of the genes KLF4, Bax and cleaved caspase-3, all of which are related to the apoptotic process and are targets of miR-367. In fact, when miR-367 expression is downregulated, Adr treatment of OS cells results in apoptosis. 

In the end, two other miRNAs (miR-140-5p and miR-184) have been identified as participating in the mechanisms of resistance to therapeutic treatments in OS. Several previous studies have demonstrated that cancer cells use autophagy to ameliorate the effects of therapeutic stress, which contribute to the development of chemoresistance [172,173]. Therefore, this ongoing consideration led Wei et al. to investigate the role of autophagy in OS cancer cells. It has been observed that OS cells treated with doxorubicin (Dox) and Cis presented an increase in miR-140-5p, which stimulates autophagy. Therefore, upregulation of miR-140-5p inhibits cell survival and chemoresistance, inducing autophagy [174]. The other miRNA involved in the chemoresistance process is miR-184, which has been studied in OS cell lines by Lin et al. They showed that treatment with Dox induces time-dependent expression of miR-184 in OS cell lines. In this study, it was observed that miR-184 reduces the number of apoptotic cells after treatment by targeting and inhibiting the BCL2L1 gene, one of the genes involved in the apoptotic process. Therefore, upregulation of miR-184 and suppression of BCL2L1 (which inhibits apoptosis) increased the resistance of OS cells to Dox. This study also proved that downregulating the expression levels of miR-184 led to an increase of Dox-induced apoptosis [175]. In conclusion, several studies have reported the discovery that miRNAs are involved in the sensitivity of OS cells to several therapeutic agents, with the final aim of understanding which molecular mechanisms confer resistance to treatments, identifying which miRNAs could be biomarkers of chemoresistance and developing targets for future therapies based on increased sensitivity to chemotherapy and radiotherapy. All the discoveries here are illustrated and summarized in Table 2.

#### 5.2.2. miRNAs and the Biology of Osteosarcoma: Latest Discoveries

Regarding the role of miRNAs in cancer progression, several studies have unravelled the dysfunction and dysregulation of miRNAs that can result in the development of OS. In identifying the latest studies on miRNAs in OS, we found that with the last year, new miRNAs involved in OS progression have been identified and could be either tumour promoters (oncomiRs) or tumour suppressors. The new oncomiRs that have been described are miR-21, miR-92b, miR-603, miR-130a, miR-488, miR-301a, miR-9 and miR-130b.

Lv et al. found that miR-21 was overexpressed in the human OS cell line MG63 [176] compared to the healthy foetal osteoblastic cell line hFOB1.19. miR-21 has been observed to be involved in regulating tumour progression and acting as a oncomiR by targeting and negatively regulating the PTEN gene, which was one of the first tumour suppressor genes identified [177]. Another miRNA related to the PTEN gene is miR-130a. An in vitro study of several types of OS cell lines revealed that upregulation of miR-130a induces cell proliferation, invasion, migration and EMT. Chen et al. also demonstrated that when PTEN is downregulated in OS, miR-130a is upregulated, and vice versa. Furthermore, restoring PTEN expression ameliorates the effects of miR-130a, demonstrating both the oncogenic role of miR-130a and its link with PTEN [178]. miR-130b, another member of the miR-130 family, is involved in OS progression similar to its activity other tumours [179,180]. 

Li et al. described that miR-130b is expressed at high levels in OS cells and promotes proliferation by binding NKD2, which leads to the suppression of Wnt signalling, the consequent inhibition of apoptosis and the promotion of proliferation [181]. The RECK gene is another tumour suppressor that is the target of the miRNA mi-92b as observed by Zhou et al. They found that miR-92b was upregulated in OS cell lines, whereas RECK was downregulated [182]. This upregulation has been reported to increase OS cell proliferation, invasion and migration by targeting the 3′-UTR of RECK and inhibiting its activity [182] by consequently blocking its ability to inhibit the metalloproteinases (MMPs) [183]. This change in activity enhances OS invasion and leads to a poor prognosis. The BRCC2 gene, a proto-oncogene expressed in several cancers, has been reported to prevent apoptosis in several cancer cell types [184,185,186]. BRCC2 is the target of the miR-603, which, as Ma et al. demonstrated, is often overexpressed in patients with OS and/or with metastases. Their study also showed that OS growth was enhanced by translational inhibition induced by the interaction between miR-603 and BRCC2, indicating that miR-603 acts as a powerful oncogene [187]. A recent report by Fenger et al. on the importance of miRNAs in OS [188] revealed that a canine model of OS demonstrated high similarity with human OS at the molecular level [189]. Furthermore, they demonstrated for the first time that miR-9 was upregulated in a canine OS cell line. miR-9 is known to perform several different functions in different cell types; for example, it can inhibit tumour growth in some cases but stimulate proliferation in others [190,191]. Fenger and colleagues also showed that in OS, miR-9 enhances cell migration and invasion, thereby promoting metastasis [188]. Their results perfectly aligned with those of recent studies regarding the upregulation of miR-9 in human OS [191]. The data obtained also paved the way to the idea that overexpression of miR-9 could be a prognostic marker of OS behaviour.

Although Fenger et al. investigated on miR-9 in OS, Ni et al. showed not only that miR-301a is overexpressed in OS cell lines but also that this miRNA enhances cell migration and inhibits apoptosis [192]. Therefore, they confirmed the carcinogenic function of miR-301a in OS, which was only observed in others cancers [193,194]. Zhou et al. investigated the activated pathways in OS cells and tissues during hypoxia. The results indicated that there is enhanced expression of miR-488 at the transcriptional level and that this upregulation promotes cell proliferation as well as reduces apoptosis and chemosensitivity. The mechanism responsible for this change in activity is the interaction of mi-488 with the predicted sequence in the 3′UTR of the Bcl-2-interacting mediator of cell death (Bim) gene [195]. All the discoveries here are illustrated and summarized in Table 3.

Next, although there are many studies that have reported the possible existence of new oncomiRs in OS, there is an increasing number of studies that have shown the first indications of new tumour suppressor miRNAs in OS. The latest tumour suppressor miRNAs in OS are miR-26a, miR-491-5p, miR-192, miR-22, miR-205, miR-506, miR-497, miR-34a, miR-203, miR-193a-3p, miR-193a-5p, miR-143, miR-182, miR-198, miR-874, miR-4262, miR-101, miR-409-3p, miR-124, miR-133a, miR-223 and miR-363. 

Li et al. investigated on miR-26a, which has been observed to be involved in osteogenesis [196] and in the differentiation of several cell types [197,198]. They demonstrated that miR-26a is downregulated OS-derived cancer stem cells (OS-CSCs) and observed that on the contrary, this upregulation decreases the expression of stem cell markers and the capacity to form sarcospheres from OS-CSCs [199]. These effects have been linked to the miR-26a target Jagged1. Jagged1 is a component of the Notch signalling pathway that, when altered, results in the loss of stemness. Therefore, miR-26a is an tumour suppressor in OS [196]. miR-491-5p has been shown to be downregulated in some tumours [200,201,202] and upregulated in others, thus, this miRNA is involved in the inhibition of tumour growth [203,204]. Yin et al. decided to investigate which role miR-491-5p plays in OS, and they reported that it is downregulated in OS cells and tissues; however, when the miR-491-5p levels were restored, they noticed that this miRNA inhibited tumour growth by targeting and negatively regulating the forkhead-box p4 (FOXP4) gene. FOXP4, a member of the FOXP subfamily, can enhance cancer proliferation and migration by targeting matrix metalloproteinase-9 (MMP9), thus acting as an oncogene [200]. This is one explanation as to why miR-491-5p is downregulated in OS—because it acts as a tumour suppressor via knockdown of FOXP4. Wang et al. decided to clarify the role of miR-192 in OS [205], which has been previously reported to be dysregulated in several cancers as well as in OS [206,207]. They reported for first time that miR-192 was downregulated in OS cells, and after transfection of a miR-192 mimic into OS cells, they observed an inhibition of cancer progression via miR-192 binding to its specific target, the TCF7 gene [205]. Wang and colleagues also demonstrated the tumour suppressor potential of miR-192 and the important link between this and the TCF7 gene, an emerging factor important not only in the inflammatory process but also in cancer [208,209]. Studying the biology of cancers has revealed that the enzyme ATP citrate lyase (ACLY) [210], a fundamental enzyme for the de novo lipogenesis [211], is usually upregulated within the tumour and that miR-22 targets this gene as shown by Xin et al. [212]. 

They reported that miR-22 could be a tumour suppressor because targeting ACLY leads to inhibition of lipid metabolism and consequent cell proliferation and migration. Additionally, miR-205 is a tumour suppressor miRNA. In fact, Zhang et al. reported that the levels of miR-205 are low in both OS tissues and cells [213]; however, elevated levels caused an inhibition of proliferation in OS cell lines. Zhang et al. also showed that one target of miR-205 is the RUNX2 gene, which is a key gene in osteogenesis [214,215]. They demonstrated that overexpression of miR-205 blocks proliferation and metastasis in OS due to knockdown of RUNX2 by miR-205 [213].

These data indicated the tumour suppressor role of miR-205. In addition, Yang et al. investigated the role of miR-205 as a tumour suppressor in OS and showed that there is a link between miR-205 and the TGF-α gene [216]. In fact, although miR-205 is downregulated in cancer, TGF-α is upregulated. Their studies have demonstrated that miR-205 targets TGF-α, which leads to an increase in the apoptosis rate by halting cells in G_0_/G_1_ phase and decreasing cell invasion [195]. Another tumour suppressor miRNA identified in OS is miR-506. Overexpression of miR-506 can suppress proliferation and enhance apoptosis by either targeting the AEG-1 gene or inhibiting the Wnt/β-catenin signalling pathway, as reported by Yao et al. [217]. They also noticed that AEG-1 could serve as a biomarker in OS because it is often elevated in patients with a poor prognosis. Ge et al. and Ruan et al. have both investigated the effects of the tumour suppressor miR-497 in OS [218,219] based on the observation that miR-497 has been observed to act as a tumour suppressor in other cancers. Ge et al. investigated the role of miR-497 in an OS cell line and in vivo using a nude mouse model and obtained similar results in both models. They demonstrated that miR-497 overexpression inhibits tumour growth and the metastatic process as well as enhances the apoptotic rate [218]. All these effects could be related to the ability of miR-497 to target several genes. However, Ruan et al. showed that the inhibitory effect of miR-497 on OS progression is related to targeting of the AMOT gene, which is very important in cell migration and cancer-induced angiogenesis, as observed in breast cancer [220]. Thus, Ruan et al. reported that the overexpression of miR-497 causes a significant decrease in angiomotin expression by negatively targeting the AMOT gene. In contrast, when miR-497 is downregulated, elevated expression of angiomotin has been observed, which is related to a poor prognosis [219].

Chen et al. investigated the activity of miR-34a and miR-203 in human OS cells [221]. First, they observed the overexpression of Survivin in OS, which has been associated with the promotion of tumour progression and metastasis. For the first time, this group showed that miR-34a and miR-203 are the only two miRNAs capable of targeting Survivin. The result of this interaction is the inhibition of the tumour progression and the induction of apoptosis and chemosensitivity [221]. Another set of tumour suppressors miRNAs includes miR-193-3p and miR-193-5p. Pu et al. indicated that the effects of these miRNAs are because they target and inhibit the Rab27b gene and the SRR gene, respectively. Both these genes appear to facilitate the invasive/metastatic phenotype of OS. Therefore, for the first time, miR-193-3p and miR-193-5p have been described as suppressors of metastasis in OS [222]. In fact, researchers have observed that when these two miRNAs are downregulated, the levels of Rab27b and SRR are no longer diminished, and the subsequent risk of metastasis is elevated, and the prognosis is poor. miR-143 is another miRNA with reduced expression in cancer. Li et al. demonstrated that the reason is because miR-143 is a tumour suppressor that targets the Bcl-2 gene, which activates the caspase-3, a pro-apoptotic enzyme [223]. Therefore, when miR-143 is upregulated, there is inhibition of OS growth. Several previous studies have identified miR-182 as an oncogene in different tumours [224,225], but Bian et al. recently demonstrated how this acts as a tumour suppressor in OS [226]. In fact, when miR-143 is upregulated, there is an increase in apoptosis and a decrease in invasion of OS. In a study on OS by Zhang et al. suggested that the observed miR-198 levels were lower than normal, which might be related to the OS stage and the presence of distant metastases [227]. In relation to this first observation, they demonstrated that overexpression of miR-198 inhibits OS cell proliferation, migration and invasion in vitro by targeting and inhibiting ROCK1 activity, which is overexpressed in OS cell lines and tissues [227]. 

Another miRNA that appears to be dysregulated in cancer is miR-874. Dong et al. investigated its role in OS and found that the restoration of miR-874 levels inhibits the OS progression by targeting E2F3, a key factor in cell cycle progression [228]. Another study showed the existence of an association between miR-4262 and osteopontin (OPN) in OS [229]. OPN was recently recognized as an important gene in the process of metastasis [230,231]. After observing that OS patients with low levels of miR-4262 had a poor prognosis, Song et al., studied miR-4262 and discovered that high levels of miR-4262 promote its activity as a tumour suppressor, and knocked down OPN, which blocked tumour invasion. In contrast, downregulation of miR-4262 prevents its targeting of OPN, thus resulting in increased OS invasion [229]. Wang et al. observed that miR-101 acts as tumour suppressor in OS by targeting c-FOS, thereby inhibiting cell proliferation [232]. Regarding the role of miR-490-3p in cancer metastasis [233,234], Wu et al. decided to investigate its role in OS metastasis [235]. Their study demonstrated that overexpression of miR-409-3p reduced OS cell migration and invasion and that this effect was mediated by targeting catenin-δ1, which has been described as an oncogene via aberrant regulation of Rho GTPases and the transcriptional activation of other oncogenes [235]. Within the last year, two groups have studied miR-124 in OS [236,237]; the first demonstrated that upregulation of miR-124, which is often downregulated in OS cells, blocks proliferation and invasion by targeting the SPHK1 gene. SPHK1 has been reported to be involved in several cellular processes including cancer metastasis. Hence, Zhou et al. have described for the first time an association between miR-124 and SPHK1 [236]. The other group showed the tumour suppressor activity of miR124 and demonstrated that this activity is related to targeting B7-H3, a protein that promotes uncontrollable proliferation and invasion [237]. Thus, Wang et al. demonstrated that elevated levels of miR124 can significantly suppress in vitro cell proliferation and invasion by targeting B7-H3 [237]. A study by Chen et al. regarding the involvement of miR-133a in OS progression indicated that miR-133a is a tumour suppressor. This is related to its association with IGF-1R, which regulates cancer cell progression and indirectly targets the AKT/ERK signalling pathway [238]. Another miRNA that has been recognized to play the role as a tumour suppressor in OS is miR-223. It has been shown that this miRNA could be a biomarker to stratify the clinical stages of OS. In fact, Dong et al. demonstrated that OS patients with lower levels of miR-223 tend to have distant metastases and a worse prognosis than OS patients with higher levels of miR-223 [239]. The most recent miRNA that was discovered to act as a tumour suppressor in OS is miR-363, which inhibits tumour progression and metastasis by targeting MAP2K4 [240]. All the discoveries here are illustrated and summarized in Table 4.

## 6. Conclusions

In summary, since the discovery of ncRNAs, researchers around the world have begun to study the effects of these molecules in human cancers. Among these, the confirmed presence of miRNAs in cancers has elevated interest in their study. Additionally, miRNAs have recently emerged as ideal molecules that could be used as diagnostic and prognostic biomarkers in many cancers. Furthermore, for rare and aggressive cancers (e.g., osteosarcoma and chondrosarcoma), miRNAs could provide a solution in the development of new therapies that will focus on either inhibiting onco-miRNAs or inducing the expression of tumour suppressor miRNAs. For this reason, many studies have been conducted regarding miRNAs in chondrosarcoma and OS. The important discoveries, which we have described in this review, show how the knowledge of miRNAs in these bone tumours is rapidly expanding. 

Although it is clear that this is the best approach in researching valid therapies for these cancers, more in-depth examinations should be conducted to go beyond the challenging problems; for example, identifying a standardized method to detect miRNAs and establishing an ideal in vivo delivery system to modulate miRNA expression. This would allow research studies on miRNAs to translate to clinical applications, with the final goal of using these targets to develop anti-cancer drugs.

## Figures and Tables

**Table 1:** Cancer-associated miRNAs in chondrosarcoma.

miRNA	Function in Chondrosarcoma	Gene Target	Reference
miR-30a	Inhibits cancer cell proliferation and invasion (Ts)	SOX4, RUNX2	[130,131,132]
miR-145	Inhibits tumour metastasis (Ts)	SOX9	[136]
miR-125b	Enhances tumour chemosensitivity (Ts)	ErbB2	[137]
miR-181a	Promotes cancer angiogenesis (Og)	VEGF	[138]
miR-126	Inhibits of cancer angiogenesis (Ts)	VCAM-1	[139]
miR-150	Promotes cancer angiogenesis (Og)	VEGF	[140]
miR-26a	Inhibits of cancer angiogenesis (Ts)	PIK3C2alpha/Akt/HIF-alpha pathway	[141]
miR-199a	Inhibits of cancer angiogenesis (Ts)	CCL5	[142]
miR-519d	Inhibits tumour metastasis (Ts)	MMP2	[144]
miR-218	Promotes COS progression (Og)	BM1	[145]
miR-185	Inhibits COS proliferation and invasion (Ts)	c-Met, DNMT1	[145]

Ts: Tumour suppressor; Og: Oncogene.

**Table 2:** Associated miRNAs to response to chemo- and radiotherapy in osteosarcoma.

miRNA	Function in Osteosarcoma—Response to Therapies	Gene Target	Reference
miR-513a-5p	Induce radiosensitivity (Ts)	APE1	[158]
miR-21	Increase cell proliferation Promote chemosensitivity to Cis (Og)	Spry1Spry2	[159]
miR-224	Inhibit cell proliferation and migration Increase sensitivity to Cis (Ts)	Rac1	[160]
miR-138	Inhibits cell proliferation and migration Increases chemosensitivity to Cis (Ts)	EZH2	[161]
miR-367	Promotes metastasis and EMT Induce chemoresistance to Adr (Og)	Bax, Cleaved Caspase-3 KLF4	[168]
miR-140-5p	Induces cell apoptosis Decreases the chemoresistance (Ts)	IP3k2	[174]
miR-184	Inhibits cell apoptosis Promotes chemosensitivity to Dox (Og)	BCL2 L1	[175]

Ts: Tumour suppressor; Og: Oncogene; EMT: Epithelial-mesenchymal transition; Cis: Cisplatin; Adr: Adriamycin; Dox: Doxorubicin.

**Table 3:** Latest discovered cancer-related oncomiRs in osteosarcoma.

miRNA	Function in Osteosarcoma	Gene Target	Reference
miR-21	Enhance the tumor progression (Og)	PTEN	[176]
miR-130a	Enhance cell proliferation and invasion Promote the EMT (Og)	PTEN	[178]
miR-130b	Enhances cancer cells doubling (Og)	NKD2	[179,180]
miR-92b	Enhance cell proliferation and invasion (Og)	RECK	[182]
miR-603	Enhance cell proliferation and invasion (Og)	BRCC2	[187]
miR-9	Enhance cell proliferation and invasion (Og)	GSN	[188,191]
miR-301a	Enhance cell proliferation and migration Inhibit apoptosis (Og)	CDC14A	[192]
miR-488	Enhance cell proliferation and migration Inhibit apoptosis Reduce chemosensitivity (Og)	Bim	[195]

Og: oncogene; EMT: Epithelial-mesenchymal transition.

**Table 4:** Latest discovered cancer related tumor suppressors miRs in osteosarcoma.

miRNA	Function in Osteosarcoma	Gene Target	Reference
miR-26a	Loss of stem like properties in CSCs (Ts)	Jagged1	[196]
miR-491-5p	Inhibit tumour growth and metastasis (Ts)	FOXP4	[200]
miR-192	Inhibit tumour progression (Ts)	TCF7	[205]
miR-22	Inhibit lipid metabolism, cell proliferation and invasion (Ts)	ACLY	[212]
miR-205	Inhibit cell proliferation and metastasis Increase of the apoptotic cell rate (Ts)	RUNX2 TGFα	[213,216]
miR-506	Inhibit cell proliferation Enhance apoptosis (Ts)	AEG-1	[217]
miR-497	Enhance apoptosis Inhibit cell proliferation and metastasis (Ts)	AMOT	[218,219]
miR-34a	Inhibit tumor progression Inhibit metastasis(Ts)	Survivin	[221]
miR-203	Inhibit tumor progression Inhibit metastasis (Ts)	Survivin	[221]
miR-193-3p	Inhibit metastasis (Ts)	Rab27B	[222]
miR-193-5p	Inhibit metastasis (Ts)	SRR	[222]
miR-143	Inhibit tumor growth (Ts)	Bcl-2	[223]
miR-182	Enhance apoptosis Inhibit invasion process (Ts)	PDCD4	[226]
miR-198	Inhibit cell proliferation and invasion (Ts)	ROCK1	[227]
miR-874	Inhibit of tumor growth (Ts)	E2F3	[228]
miR-4262	Inhibit tumor invasion (Ts)	OPN	[229]
miR-101	Inhibit cell proliferation and invasion (Ts)	c-FOS	[232]
miR-409-3p	Inhibit cell invasion and metastasis (Ts)	Rho GTPases	[235]
miR-124	Inhibit cell proliferation and invasion (Ts)	SPHK1 B7-H3	[236,237]
miR-133a	Inhibit tumor growth (Ts)	AKT/ERK signaling pathway	[238]
miR-223	Inhibit cell invasion (Ts)	Ect2	[239]
miR-363	Inhibit cell growth and metastasis (Ts)	MAP2K4	[240]

Ts: Tumor suppressor; CSCs: Cancer stem cells.

## References

[1] Chen X., Fan S., Song E., Song E. (2016). Noncoding RNAs: New players in cancers. The Long and Short Non-Coding RNAs in Cancer Biology.

[2] Lee R.C., Feinbaum R.L., Ambros V. (1993). The *C. elegans* heterochronic gene lin-4 encodes small RNAs with antisense complementarity to lin-14. Cell.

[3] Hwang H.W., Mendell J.T. (2006). MicroRNAs in cell proliferation, cell death, and tumorigenesis. Br. J. Cancer.

[4] Ebert M.S., Sharp P.A. (2012). Roles of microRNAs in conferring robustness to biological processes. Cell.

[5] Fire S., Xu S., Montgomery M.K., Kostas S.A., Driver S.E., Mello C.C. (1998). Potent and specific genetic interference by double-stranded RNA in *Caenorhabditis elegans*. Nature.

[6] Reinhart B.J., Slack F.J., Basson M., Pasquinelli A.E., Bettinger J.C., Rougvie A.E., Horvitz H.R., Ruvkun G. (2000). The 21-nucleotide let-7 RNA regulates developmental timing in *Caenorhabditis elegans*. Nature.

[7] Ambros V. (2004). The function of animals microRNAs. Nature.

[8] Lagos-Quintana M., Rauhut R., Lendeckel W., Tuschl T. (2001). Identification of novel genes coding for small expressed RNAs. Science.

[9] Grishok A., Paquinelli A.E., Conte D., Li N., Parrish S., Ha I., Baillie D.L., Fire A., Ruvkun G., Mello C.C. (2001). Genes and mechanisms related to RNA interference regulate expression of the small temporal RNAs that control C. elegans developmental timing. Cell.

[10] Sharp P.A. (2001). RNA interference. Genes Dev..

[11] Bartel D.P. (2004). MicroRNAs: Genomic, biogenesis, mechanism, and function. Cell.

[12] Hansen T.B., Jensen T.I., Clausen B.H., Bramsen J.B., Finsen B., Damgaard C.K., Kjems J. (2013). Natural RNA circles function is efficient microRNA sponges. Nature.

[13] Diederichs S. (2014). The four dimensions of noncoding RNA conservation. Trends Genet..

[14] Galupa R., Heard E. (2015). X-chromosome inactivation: New insights into *cis* and *trans* regulation. Curr. Opin. Genet. Dev..

[15] Rinn J.L., Kertesz M., Wang J.K., Squazzo S.L., Xu X., Brugmann S.A., Goodnough L.H., Helms J.A., Farnham P.J., Segal E. (2007). Functional demarcation of active and silent chromatin domains in human HOX loci by noncoding RNAs. Cell.

[16] Liz J., Esteller M. (2016). LncRNAs and microRNAs with the role in cancer development. Biochim. Biophys. Acta.

[17] Cabili M.N., Trapnell C., Goff L., Koziol M., Tazon-Vega B., Regev A., Rinn J.L. (2011). Integrative annotation of human large intergenic non-coding RNAs reveals global properties and specific subclasses. Genes Dev..

[18] Huang T., Alvarez A., Hu B., Cheng S.Y. (2013). Noncoding RNAs in cancer and cancer stem cells. Chin. J. Cancer.

[19] Kapranov P., Cheng J., Dike S., Nix D.A., Duttagupta R., Willingham A.T., Stadler P.F., Hertel J., Hackermuller J., Hofacker I.L. (2007). RNA maps reveal new RNA classes and a possible function for pervasive transcription. Science.

[20] Huarte M., Guttman M., Feldser D., Garber M., Koziol M.J., Kenzelmann-Broz D., Khalil A.M., Zuk O., Amit I., Rabani M. (2010). A large intergenic noncoding RNA induced by p53 mediates global gene repression in the p53 response. Cell.

[21] Heo J.B., Sung S. (2011). Vernalization-mediated epigenetic silencing by a long intronic noncoding RNA. Science.

[22] Kim T.-H., Hemberg M., Gray J.M., Costa A.M., Bear D.M., Wu J., Harmin D.A., Laptewicz M., Barbara-Haley K., Kuersten S. (2011). Widespread transcription at neuronal activity enhancers. Nature.

[23] Tripathi V., Ellis J.D., Shen Z., Song D.Y., Pan Q., Watt A.T., Freier S.M., Bennett C.F., Sharma A., Bubulya P.A. (2010). The nuclear-retained noncoding RNA MALAT1 regulates alternative splicing by modulating SR splicing factor phosphorylation. Mol. Cell..

[24] Mahmoudi S., Henriksson S., Corcoran M., Mendez-Vidal C., Wiman K.G., Farnebo M. (2016). Wrap53, a natural p53 antisense transcript required for p53 induction upon DNA damage. Mol. Cell.

[25] Novikova I.V., Hennelly S.P., Tung C.S., Sanbonmatsu K.Y. (2013). Rise of the RNA machines: Exploring the structure of long non-coding RNAs. J. Mol. Biol..

[26] Nagano T., Mitchell J.A., Sanz L.A., Pauler F.M., Ferguson-Smith A.C., Feil R., Fraser P. (2008). The air noncoding RNA epigenetically silences transcription by targeting G9a to chromatin. Science.

[27] Ingolia N.T., Lareau L.F., Weissman J.S. (2011). Ribosome profiling of mouse embryonic stem cells reveals the complexity and dynamics of mammalian proteomes. Cell.

[28] Karreth F.A., Tay Y., Perna D., Ala U., Tan S.M., Rust A.G., DeNicola G., Webster K.A., Weiss D., Perez-Mancera P.A. (2011). In vivo identification of tumor-suppressive PTEN ceRNAs in an oncogenic BRAF-induced mouse model melanoma. Cell.

[29] Johnsson P., Ackley A., Vidarsdottir L., Lui W.O., Corcoran M., Grandér D., Morris K.V. (2013). A pseudogene long-noncoding-RNA network regulates PTEN transcription and translation in human cells. Nat. Struct. Mol. Biol..

[30] Poliseno L., Salmena L., Zhang J., Carver B., Haveman W.J., Pandolfi P.P. (2010). A coding-independent function of gene and pseudogene mRNAs regulates tumor biology. Nature.

[31] Hung T., Chang Y. (2010). Long non-coding RNA in genome regulation prospects and mechanisms. RNA Biol..

[32] Van Leeuwen S., Mikkers H. (2010). Long non-coding RNAs: Guardians of development. Differentiation.

[33] Zhang Y., Zou Y., Wang W., Zuo Q., Jiang Z., Sun M., De W., Sun L. (2015). Down-regulated long non-coding RNA MEG3 and its effect on promoting apoptosis and suppressing migration of trophoblast cells. J. Cell Biochem..

[34] Fang Y., Fullwood J.M. (2016). Roles, functions and mechanisms of long non-coding RNAs in cancer. Genomics Proteom. Bioinform..

[35] Deng H., Zhang J., Shi J., Guo Z., He C., Ding L., Tang J.H., Hou Y. (2016). Role of long non-coding RNA in tumor drug resistance. Tumour Biol..

[36] Pichler M., Calin G.A. (2015). MicroRNAs in cancer: From developmental genes in worms to their clinical application in patients. Br. J. Cancer.

[37] Lin N., Chang K.Y., Li Z., Gates K., Rana Z.A., Dang J., Zhang D., Han T., Yang C.S., Cunningham T.J. (2014). An evolutionarily conserved long non-coding RNA Tuna controls pluripotency and neural lineage commitment. Mol. Cell.

[38] Klattenhoff C.A., Scheuermann J.C., Surface L.E., Bradley R.K., Fields P.A., Steinhauser M.L., Ding H., Butty V.L., Torrey L., Haas S. (2013). Braveheart, a long non-coding RNA required for cardiovascular lineage commitment. Cell.

[39] Wang K., Liu F., Zhou L.Y., Long B., Yuan S.M., Wang Y., Liu C.Y., Sun T., Zhang X.J., Li P.F. (2014). The long non-coding RNA CHRF regulates cardiac hypertrophy by targeting miR-489. Circ. Res..

[40] Roderburg C., Urban G.W., Bettermann K., Vucur M., Zimmermann H., Schmidt S., Janssen J., Koppe C., Knolle P., Castoldi M. (2011). Micro-RNA profiling reveals a role for miR-29 in human murine liver fibrosis. Hepatology.

[41] Thum T. (2014). Non-coding RNAs and myocardial fibrosis. Nat. Rev. Cardiol..

[42] Bayoumi A.S., Sayed A., Broskova Z., Teoh J.-P., Wilson J., Su H., Tang Y.-L., Kim I.M. (2016). Crosstalk between long noncoding RNAs and microRNAs in health and disease. Int. J. Mol. Sci..

[43] Su X., Xing J., Wang Z., Chen L., Cui M., Jiang B. (2013). microRNAs and ceRNAs: RNA networks in pathogenesis of cancer. Chin. J. Cancer Res..

[44] Chen C.L., Tseng Y.W., Wu J.C., Chen G.Y., Lin K.C., Hwang S.M., Hu Y.C. (2015). Suppression of hepatocellular carcinoma by baculovirus-mediated expression of long non-coding RNA PTENP1 and microRNA regulation. Biomaterials.

[45] Shi S.J., Wang L.J., Yu B., Li Y.H., Jin Y., Bai X.Z. (2015). LncRNA-ATB promotes trastuzumab resistance and invasion-metastases cascade in breast cancer. Oncotarget.

[46] Chiyomaru T., Yamamura S., Fukuhara S., Yoshino K., Kinoshita T., Majid S., Saini S., Chang I., Tanaka Y., Enokida H. (2013). Genistein inhibits prostate cancer cell growth by targeting miR-34a and oncogenic HOTAIR. PLoS ONE.

[47] Khorshidi A., Dhaliwal P., Burton B.Y., Song E. (2016). Noncoding RNA in tumour angiogenesis. The Long and Short Non-Coding RNAs in Cancer Biology.

[48] Leucci E., Patella F., Waagee J., Holmstrøm K., Lindow M., Porse B., Kauppinen S., Lund A.H. (2013). MicroRNA-9 targets the long non-coding RNA MALAT1 for degradation in the nucleus. Sci. Rep..

[49] Bentwich I., Avniel A., Karov Y., Aharonov R., Gilad S., Barad O., Barzilai A., Einat P., Einav U., Meiri E. (2005). Identification of hundreds of conserved and nonconserved human microRNAs. Nat. Genet..

[50] Calin G.A., Dimitru C.D., Shimizu M., Bichi R., Zupo S., Noch E., Aldler H., Rattan S., Keating M., Rai K. (2002). Frequent deletions and down-regulation of micro-RNA genes miR15 and miR16 at 13q14in chronic lymphocytic leukemia. Proc. Natl. Acad. Sci. USA.

[51] Farazi T.A., Spitzer J.I., Morozov P., Tuschl T. (2011). MicroRNAs in human cancer. J. Pathol..

[52] Costa P.M., Pedroso de Lim M.C. (2013). MicroRNAs as molecular targets for cancer therapy: On the modulation of microRNA expression. Pharmaceuticals (Basel).

[53] Hayes J., Peruzzi P.P., Lawler S. (2014). MicroRNAs in cancer: Biomarkers, functions and therapy. Trends Mol. Med..

[54] Bielack S.S., Kempf-Bielack B., Delling G., Exner G.U., Flege S., Helmeke K., Kotz R., Salzer-Kuntschik M., Werner M., Winkelmann W. (2002). Prognostic factors in high-grade osteosarcoma of the extremities or trunk: An analysis of 1702 patients treated on neoadjuvant cooperative osteosarcoma group protocols. J. Clin. Oncol..

[55] Pasquinelli A.E., Hunter S., Bracht J. (2005). MicroRNAs: A developing story. Curr. Opin. Genet. Dev..

[56] Alvarez-Garcia I., Miska A.E. (2005). Microrna function in animal development and human disease. Development.

[57] Klattenhoff C., Theurkauf W. (2008). Biogenesis and germline functions of piRNA. Development.

[58] Ghildiyal M., Seitz H., Horwich M.D., Li C., Du T., Lee S., Xu J., Kittler E.L., Zapp M.L., Weng Z. (2008). Endogenous siRNA derived from transposons and mRNAs in *Drosophila* somatic cell. Science.

[59] Lee R.C., Ambros V. (2001). An extensive class of small RNAs in Caenorhabditis elegans. Science.

[60] Griffith-Jones S., Saini H.K., van Dongen S., Enright A.J. (2008). miRBase: Tools for microRNA genomics. Nucleic Acid Res..

[61] Friedman R.C., Farh K.K., Burge C.B., Bartel D.P. (2009). Most mammalian mRNAs are conserved targets of microRNAs. Genome Res..

[62] Ruby J.G., Jan C.H., Bartel D.P. (2007). Intronic microRNA precursors that bypass Drosha processing. Nature.

[63] Cifuentes D., Xue H., Taylor D.W., Patnode H., Mishima Y., Cheloufi S., Ma E., Mane S., Hannon G.J., Lawson N.D. (2010). A novel miRNA processing pathway independent of Dicer requires Argonaute2 catalytic activity. Science.

[64] Lee Y., Jeon K., Lee J.T., Kim S., Kim V.N. (2002). microRNA maturation: Stepwise processing and subcellular localization. EMBO J..

[65] Yi R., Qin Y., Macara I.G., Cullen B.R. (2003). Exportin-5 mediates the nuclear export of premicroRNAs and short hairpin RNAs. Gene Dev..

[66] Han J., Pedersen J.S., Kwon S.C., Belair C.D., Kim Y.K., Yeom K.H., Yang W.Y., Haussler D., Blelloch R., Kim V.N. (2009). Posttranscriptional cross regulation between Drosha and DGCR8. Cell.

[67] Triboulet R., Chang H.M., Lapierre R.J., Gregory R.I. (2009). Post-transcriptional control of DGCR8. RNA.

[68] Lagos-Quintana M., Rauhut R., Yalcin A., Meyer J., Lendeckel W., Tuschl T. (2002). Identification of tissue-specific microRNAs from mouse. Curr. Biol..

[69] Kosik K.S. (2006). The neuronal microRNA system. Nat. Rev..

[70] Li M.A., He L. (2012). microRNAs as novel regulators of stem cell pluripotency and somatic cell reprogramming. BioEssays.

[71] Raghu R., Mendell J.T. (2009). Abate and switch: MiR-145 in stem cell differentiation. Cell.

[72] Houbaviy H.B., Murray M.F., Sharp P.A. (2003). Embrionic stem cells specific microRNAs. Dev. Cell.

[73] Xu N., Papagiannakopoulos T., Pan G., Thompson J.A., Kosik K.S. (2009). MicroRNA-145 regulates OCT4, SOX2, and KLF4 and represses pluripotency in human Embryonic Stem Cells. Cell.

[74] Zovoillis A., Smorag L., Patanzi A., Engel W. (2009). Members of the miR-290 cluster modulate in vitro differentiation of mouse embryonic stem cells. Differentiation.

[75] Schultz J., Lorenz P., Gross G., Ibrahim S., Kunz M. (2008). MicroRNA let-7b targets important cell cycle molecules in malignant melanoma cells and interferes with anchorage-independent growth. Cell Res..

[76] Flemming A. (2014). Heart failure: Targeting miRNA pathology in heart disease. Nat. Rev. Drug Discov..

[77] Yu F., Lu Z., Cai J., Huang K., Chen B., Li G., Dong P., Zheng J. (2015). MALAT1 functions as a competing endogenous RNA to mediate Rac1 expression by sequestering miR-101b in liver fibrosis. Cell Cycle.

[78] Farr R.J., Joglekar M.V., Hardikar A.A. (2015). Circulating microRNAs in diabetes progression: Discovery, validation, and research translation. EXS.

[79] Alageel A.M., Abou Al-Shaar H., Shariff R.K., Albakr A. (2016). The role of RNA metabolism in neurological diseases. Balkan J. Med. Genet..

[80] Xi J.J. (2013). MicroRNAs in cancer. Cancer Treat. Res..

[81] Mandel P., Metais P. (1948). Les acides nucléics du plasma sanguine chez l’homme. C. R. Seances Soc. Biol. Fil..

[82] Tan E.M., Schur P.H., Carr R.I., Kunkel H.G. (1966). Deoxybonucleic acid (DNA) and antibodies to DNA in the serum of patients with systemic lupus erythematosus. J. Clin. Investig..

[83] Leon S.A., Shapiro B., Sklaroff D.M., Yaros M.J. (1977). Free DNA in the serum of cancer patients and the effect of therapy. Cancer Res..

[84] Lawrie C.H., Gal S., Dunlop H.M., Pushkaran B., Liggins A.P., Pulford K., Banham A.H., Pezzella F., Boultwood J., Wainscoat J.S. (2008). Detection of elevated levels of tumor-associated micro-RNAs in serum of patients with diffuse large-B-cell lymphoma. Br. J. Haematol..

[85] Corsten M.F., Dennert R., Jochems S., Kuznetsova T., Deveaux Y., Hofstra L., Wagner D.R., Staessen J.A., Heymans S., Schroen B. (2010). Circulating microRNA-208b and microRNA-499 reflect myocardial damage in cardiovascular disease. Circ. Cardiovasc. Genet..

[86] Turkinovich A., Weisz L., Burwinkel B. (2012). Extracellular miRNAs: The mystery of their origin and function. Trends Biochem. Sci..

[87] Turkinovich A., Weisz L., Langheinz A., Burwinkle B. (2011). Characterization of extracellular circulating microRNA. Nucleic Acids Res..

[88] Lakkaraju A., Rodriguez-Boulan E. (2008). Itinerant exosomes: Emerging roles in cell and tissue polarity. Trends Cell Biol..

[89] Valadi H., Ekstrom K., Bossios A., Sjostrand M., Lee J.L., Lötvall J.O. (2007). Exosome-mediated transfer of mRNA and microRNAs is a novel mechanism of genetic exchange between cells. Nat. Cell Biol..

[90] Hunter M.P., Ismail N., Zhang X., Aguda B.D., Lee E.J., Yu L., Xiao T., Shafer J., Lee M.L., Schmittgen T.D. (2008). Detection of microRNA expression in human peripheral blood microvesicles. PLoS ONE.

[91] Wittman J., Jäck H.M. (2010). Serum microRNAs as powerful cancer biomarkers. Biochim. Biophys. Acta.

[92] Larrea E., Sole C., Manterola L., Goiocoechea I., Armesto M., Arestin M., Caffarel M.M., Araujo A.M., Araiz M., Ferandez-Mercado M. (2016). New concepts in cancer biomarkers: Circulating miRNAs in liquid biopsies. Int. J. Mol. Sci..

[93] De Planell-Saguer M., Rodicio M.C. (2013). Detection methods for microRNAs in clinic practice. Clin. Biochem..

[94] Chen C., Ridzon D.A., Broomer A.J., Zhou Z., Lee D.H., Nguyen J.T., Barbisin M., Xu N.L., Mahuvakar V.R., Andersen M.R. (2005). Real-time quantification of microRNAs by stem-loop RT-PCR. Nucleic Acids Res..

[95] Schmittgen T.D., Lee E.J., Jiang J., Sarkar A., Yang L., Elton T.S., Chen C. (2008). Real-time PCR quantification of precursor and mature microRNA. Methods.

[96] Schmittgen T.D., Jiang J., Liu Q., Yang L. (2005). A high-throughput method to monitor the expression of microRNA precursors. Nucleic Acids Res..

[97] Nelson P.T., Baldwin D.A., Kloosterman W.P., Kauppinen S., Plasterk R.H., Mourelats Z. (2006). RAKE and LNA-ISH reveal microRNA expression and localization in archival human brain. RNA.

[98] Hanna J.A., Wimberly H., Kumar S., Slack F., Agarwal S., Rimm D.L. (2012). Quantitative analysis of microRNAs in tissue microarrays by in situ hybridization. Biotechniques.

[99] Li W., Ruan K. (2009). MicroRNA detection by microarray. Anal. Bioanal. Chem..

[100] Khan N., Mironov G., Berezovski M.V. (2016). Direct detection of endogenous MicroRNA and their post-transcriptional modifications in cancer serum by capillary electrophoresis-mass spectrometry. Anal. Bioanal. Chem..

[101] Roy S., Soh J.H., Ying J.Y. (2016). A microarray platform for detecting disease-specific circulating miRNA. Biosens. Bioelectron..

[102] Ren Y., Deng H., Shen W., Gao Z. (2013). A highly sensitive and selective electrochemical biosensor for direct detection of MicroRNAs in serum. Anal. Chem..

[103] Ferracin M., Lupini L., Mangolini A., Negrini M. (2016). Circulating Non-coding RNA as biomarkers in colorectal cancer. Adv. Exp. Mol. Biol..

[104] Pencheva N., Tavazoie S.F. (2013). Control of metastatic progression by microRNA regulatory networks. Nat. Cell Biol..

[105] Novello C., Pazzaglia L., Cingolani C., Conti A., Quattrini I., Manara M.C., Tognon M., Picci P., Benassi M.S. (2013). miRNA expression profile in human osteosarcoma: Role of miR-1 and miR-133b in proliferation and cell cycle control. Int. J. Oncol..

[106] Xu L., Zhang Y., Wang H., Zhang G., Ding Y., Zhao L. (2014). Tumor suppressor miR-1 restrains epithelial-mesechymal transition and metastasis of colorectal carcinoma via the MAPK and PI3K/AKT pathway. J. Trans. Med..

[107] Wang T., Yuan J., Feng N., Li Y., Lin Z., Jiang Z., Gui Y. (2014). Hsa-miR-1 downregulates long non-coding RNA urothelial cancer associated 1 in bladder cancer. Tumour. Biol..

[108] Boyerinas B., Park S.M., Hau A., Murmann A.E., Peter M.E. (2010). The role of let-7 in cell differentiation and cancer. Endocr. Relat. Cancer.

[109] Ma L., Teruya-Feldestein J., Weinberg R.A. (2007). Tumor invasion and metastasis initiated by microRNA-10b in breast cancer. Nature.

[110] Li J., Fu R., Yang L., Tu W. (2015). miR-21 expression predicts prognosis in diffuse large B-cell lymphoma. Int. J. Clin. Exp. Pathol..

[111] Mashima R. (2015). Physiological roles of miR-155. Immunology.

[112] Hong Q., Fang J., Pang Y., Zheng J. (2014). Prognostic value of microRNA-29 family in patients with primary osteosarcomas. Med. Oncol..

[113] Bader A.G. (2012). miR34—A microRNA replacement therapy is headed to the clinic. Front. Genet..

[114] Li Z., Hassan M.Q., Volinia S., van Wijnen A.J., Stein J.L., Croce C.M., Lian J.B., Stein G.S. (2008). A microRNA signature for a BMP2-induced osteoblast lineage commitment program. Proc. Natl. Acad. Sci. USA.

[115] Peng S., Gao D., Gao C., Wei P., Niu M., Shuai C. (2016). MicroRNAs regulate signaling pathways in osteogenic differentiation of mesenchymal stem cells. Mol. Med. Rep..

[116] Logan C.Y., Nusse R. (2004). The Wnt signaling pathway in development and disease. Annu. Rev. Cell Dev. Biol..

[117] Sun F., Wan M., Xu X., Gao B., Zhou Y., Sun J., Cheng L., Klein O.D., Zhou X., Zheng L. (2014). Crosstalk between miR-34a and Notch Signaling Promotes Differentiation in Apical Papilla Stem Cells (SCAPs). J. Dent. Res..

[118] Yang N., Wang G., Hu C., Shi Y., Liao L., Shi S., Cai Y., Cheng S., Wang X., Liu Y. (2013). Tumor necrosis factor α suppresses the mesenchymal stem cell osteogenesis promoter miR-21 in estrogen deficiency-induced osteoporosis. J. Bone Miner Res..

[119] Inose H., Ochi H., Kimura A., Fujita K., Xu R., Sato S., Iwasaki M., Sunamura S., Takeuchi Y., Fukumoto S. (2009). A microRNA regulatory mechanism of osteoblast differentiation. Proc. Natl. Acad. Sci. USA.

[120] Luzi E., Marini F., Sala S.C., Tognarini I., Galli G., Brandi M.L. (2008). Osteogenic differentiation of human adipose tissue-derived stem cells is modulated by the miR-26a targeting of the SMAD1 transcription factor. J. Bone Miner Res..

[121] Kim Y.J., Bae S.W., Yu S.S., Bae Y.C., Jung J.S. (2009). miR-196a regulates proliferation and osteogenic differentiation in mesenchymal stem ce4lls derived from human adipose tissue. J. Bone Miner Res..

[122] Ji X., Chen X., Yu X. (2016). MicroRNAs in osteoclastogenesis and function: Potential therapeutic targets for osteoporosis. Int. J. Mol. Sci..

[123] Franceschetti T., Kessler C.B., Lee S.K., Delany A.M. (2013). miR-29 promotes murine osteoclastogenesis by regulating osteoclast commitment and migration. J. Cell Biochem..

[124] Sugatani T., Hruska K.A. (2007). MicroRNA-223 is a key factor in osteoclast differentiation. J. Biol. Chem..

[125] Tuddenham L., Wheeler G., Ntounia-Fousara S., Waters J., Hajihosseini M.K., Clark I., Dalmay T. (2006). The cartilage specific microRNA-140 targets histone deacetylase 4 in mouse cells. FEBS Lett..

[126] Martinez-Sanchez A., Dudek K.A., Murphy C.L. (2012). Regulation of human chondrocyte function through direct inhibition of cartilage master-regulator SOX9 by miR-145. J. Biol. Chem..

[127] Nugent M., Santulli G. (2015). microRNA and bone cancer. microRNA: Cancer.

[128] Leddy L.R., Holmes R.E. (2014). Chondrosarcoma of bone. Cancer Treat. Res..

[129] Frezza A.M., Cesari M., Baumhoer D., Biau D., Bielack S., Campanacci D.A., Casanova J., Esler C., Ferrari S., Funovics P.T. (2015). Mesenchymal chondrosarcoma: Prognostic factors and outcome in 113 patients. A European Musculoskeletal Oncology Society study. Eur. J. Cancer.

[130] Lu N., Lin T., Wang L., Qi M., Liu Z., Dong H., Zhang X., Zhai C., Wang Y., Liu L. (2015). Association of SOX4 regulated by tumor suppressor miR-30a with poor prognosis in low-grade chondrosarcoma. Tumor Biol..

[131] Akiyama H., Chaboisser M.C., Martin J.F., Schedl A., de Crombugghe B. (2002). The transcription factor Sox9 has essential roles in successive steps of the chondrocyte differentiation pathway and is required for expression of Sox5 and Sox6. Genes Dev..

[132] Jiang D., Zheng X., Shan W., Shan Y. (2016). The overexpression of miR-30a affects cell proliferation of chondrosarcoma via targeting Runx2. Tumor Biol..

[133] Kamachi Y., Kondoh H. (2013). Sox proteins: Regulators of cell fate specification and differentiation. Development.

[134] Ikegami D., Akiyama H., Suzuki A., Nakamura T., Nakano T., Yoshikawa H., Tsumaki N. (2011). Sox9 sustains chondrocyte survival and hypertrophy in part through Pik3ca-Akt pathways. Development.

[135] Power P.F., Mak I.W., Singh S., Popovic S., Gladdy R., Ghert M. (2001). ETV5 as a regulator of matrix metalloproteinase 2 in human chondrosarcoma. J. Orthop. Res..

[136] Mak I.W.Y., Singh S., Turcotte R., Ghert M. (2015). The epigenetic regulation of SOX9 by miR-145 in human chondrosarcoma. J. Cell Biochem..

[137] Tang X., Zheng W., Ding M., Guo K., Feng H., Deng B., Hou Y., Gao L. (2016). miR-125b acts as tumor suppressor in in chondrosarcoma cells by the sensitization to doxoribucin through direct targeting the ErbB2-regulated glucose metabolism. Drugs Des. Dev. Ther..

[138] Sun X., Wei L., Chen Q., Terek R.M. (2015). MicroRNA regulates vascular endothelial growth factor expression in chondrosarcoma cells. Clin. Orthop. Relat. Res..

[139] Liu B., Peng X.C., Zheng X.L., Wang J., Qin Y.W. (2009). MiR-126 restoration down-regulate VEGF and inhibit the growth of lung cancer cell lines in vitro and in vivo. Lung Cancer.

[140] Liu Y., Zhao L., Li D., Yin Y., Zhang Y. (2013). Microvescicle-delivery miR-150 promotes tumorigenesis by upregulation VEGF, and the neutralization of miR-150 attenuate tumor development. Protein Cell.

[141] Chai Z.T., Kong J., Zhu X.D., Zhang Y.Y., Lu L., Zhou J.M., Wang L.R., Zhang K.Z., Zhang Q.B., Ao J.Y. (2013). MicroRNA-26a inhibits angiogenesis by downregulating VEGFA through the PIK3C2α/Akt/HIF-1α pathway in hepatocellular carcinoma. PLoS ONE.

[142] Liu G.-T., Huang Y.-L., Tzeng H.-E., Tsai C.-H., Wang S.-W., Tang C.-H. (2015). CCL5 promotes vascular endothelial growth factor expression and induces angiogenesis by down-regulating miR-199a in human chondrosarcoma cells. Cancer Lett..

[143] Stetler-Stevenson W.G. (2001). The role of matrix metalloproteinases in tumor invasion, metastasis, and angiogenesis. Surg. Oncol. Clin. N. Am..

[144] Tsai C.-H., Tsai H.-C., Huang N.-H., Hung C.-H., Hsu C.-J., Fong Y.-C., Hsu H.-C., Huang Y.-L., Tang C.-H. (2015). Resistin promotes tumor metastasis by down-regulation of miR-519d through the AMPK/p38 signaling pathway in human chondrosarcoma cells. Oncotarget.

[145] Goudarzi P.K., Taheriazam A., Asghari S., Jamshidi M., Shakeri M., Yahaghi E., Mirghasemi A. (2016). Downregulation of miR-185 and uperegulation of miR-218 expression may be potential diagnostic and prognostic biomarkers of human chondrosarcoma. Tumor Biol..

[146] Xiang Y., Ma N., Wang D., Zhang Y., Zhou J., Wu G., Zhao R., Huang H., Wang X., Qiao Y. (2014). MiR-152 and MiR-185 co-contribute to ovarian cancer cells cisplatin sensitivity by targeting DNMT1 directly; a novel epigenetic therapy independent of decitabine. Oncogene.

[147] Fu P., Du F., Yao M., Lv K., Liu Y. (2014). microRNA-185 inhibits proliferation by targeting c-Met in human breast cancer cells. Exp. Ther. Med..

[148] Tian H., Hou L., Xiong Y.M., Huang J.X., She Y.J., Bi X.B. (2015). MiR-218 suppresses tumor growth and enhances the chemosensitivity of esophageal squamous cell carcinoma to cisplatin. Oncol. Rep..

[149] Ottaviani G., Jaffe N. (2009). The epidemiology of osteosarcoma. Cancer Treat. Res..

[150] Bacci G., Briccoli A., Rocca M., Ferrari S., Donati D., Longhi A., Bertoni F., Bacchini P., Giacomini S., Forni C. (2003). Neoadjuvant chemotherapy for osteosarcoma of the extremities with metastases at presentation: Recent experience at the Rizzoli Insitute in 57 patients treated with cisplatin, doxorubicin, and a high dose of methotrexate and ifosfamide. Ann. Oncol..

[151] Ferguson W.S., Goorin A.M. (2001). Current treatment of osteosarcoma. Cancer Investig..

[152] Yu X., Li Z., Yu J., Chasn M.T.V., Wu W.K.K. (2015). MicroRNAs predict and modulate responses to chemotherapy in colorectal cancer. Cell Prolif..

[153] Song B., Wang Y., Xi Y., Kudo K., Bruheim S., Botchkina G.I., Gavin E., Wan Y., Formentini A., Kormann M. (2009). Mechanism of chemoresistance mediated by miR-140 in human osteosarcoma and colon cancer cells. Oncogene.

[154] Mueller A.K., Lindner K., Hummel R., Haier J., Watson D.I., Hussey D.J. (2016). MicroRNAs and their impact on Radiotherapy for cancer. Radiat. Res..

[155] Naidu M.D., Mason J.M., Pica R.V., Fung H., Pena L.A. (2010). Radiation resistance in glioma cells determined by DNA damage repair activity of Ape1/Ref-1. J. Radiat. Res..

[156] Sak S.C., Harnden P., Johnston C.F., Paul A.B., Kiltie A.E. (2005). APE1 and XRCC1 protein expression levels predict cancer-specific survival following radical radiotherapy in bladder cancer. Clin. Cancer Res..

[157] Hong J., Chen Z., Peng D., Zaika A., Revetta F., Washington M.K., Belkhiri A., El-Rifai W. (2016). APE1-mediated DNA damage repair provides survival advantage for esophageal adenocarcinoma cells in response to acidic bile salts. Oncotarget.

[158] Dai N., Qing Y., Cun Y., Zhong Z., Li C., Zhang S., Shan J., Yang X., Dai X., Cheng Y. (2016). miR-513a-5p regulates radiosensitivity of osteosarcoma by targeting human apurinic/apyrimidinic endonuclease. Oncotarget.

[159] Vanas V., Haigi B., Stockhammer V., Sutterlüty-Fall H. (2016). MicroRNA-21 increases proliferation and cisplatin sensitivity of osteosarcoma derived cell. PLoS ONE.

[160] Geng S., Gu L., Ju F., Zhang H., Wang Y., Tang H., Bi Z., Yang C. (2016). MicroRNA-224 promotes the sensitivity of osteosarcoma cells to cisplatin by targeting Rac1. J. Cell Mol. Med..

[161] Zhu Z., Tang J., Wang J., Duan G., Zhou L., Zhou X. (2016). MiR-138 acts a tumor suppressor by targeting EZH2 and enhances cisplatin-induced apoptosis in osteosarcoma cells. PLoS ONE.

[162] Slaby O., Svobova M., Fabian P., Smerdova T., Knoflickova D., Bednarikova M., Nenutil R., Vyzula R. (2007). Altered expression of miR-21, miR-31, miR-143 and miR-145 is related to clinicopathologic features of colorectal cancer. Oncology.

[163] Yanaihara N., Caplen N., Bowman E., Seike M., Kumamoto K., Yi M., Stephens R.M., Okamoto A., Yokota J., Tanaka T. (2006). Unique microRNA molecular profiles in lung cancer diagnosis and prognosis. Cancer Cell..

[164] Iorio M.V., Ferracin M., Liu G.G., Veronese A., Spizzo R., Sabbioni S., Magri E., Pedriali M., Fabbri M., Campiglio M. (2005). MicroRNA gene expression deregulation in human breast cancer. Cancer Res..

[165] Meng F., Henson R., Wehbe-Janek H., Ghoshal K., Jacob S.T., Patel T. (2007). MicroRNA-21 regulates expression of the PTEN tumor suppressor gene in human hepatocellular cancer. Gastroenterology.

[166] Folini M., Gandellini P., Longoni N., Profumo V., Callari M., Pennati M., Colecchia M., Supino R., Veneroni S., Salvioni R. (2010). miR-21: An oncomir on strike in prostate cancer. Mil. Cancer.

[167] Yang S.M., Huang C., Li X.F., Yu M.Z., He Y., Li J. (2016). miR-21 confers cisplatin resistance in gastric cancer cells by regulating PTEN. PLoS ONE.

[168] Wang G.C., He Q.Y., Tong D.K., Wang C.F., Liu K., Ding C., Ji F., Zhang H. (2016). MiR-367 negatively regulates apoptosis induced by adriamycin in osteosarcoma cells by targeting KLF4. J. Bone Oncol..

[169] Zhu Z., Xu Y., Zhao J., Liu Q., Feng W., Fan J., Wang P. (2015). miR-367 promotes epithelial-to-mesenchymal transition and invasion of pancreatic ductal adenocarcinoma cells by targeting the Smad7-TGF-β signalling pathway. Br. J. Cancer.

[170] Chae Y.S., Kim J.G., Kang B.W., Lee S.J., Lee Y.J., Park J.S., Choi G.S., Lee W.K., Jeon H.S. (2013). Functional polymorphism in the MicroRNA-367 binding site as a prognostic factor for colonic cancer. Anticancer Res..

[171] Zhang B., He D., Fang X., Xu H., Yang Q. (2015). The microRNA-367 inhibits the invasion and metastasis of gastric cancer by directly repressing Rab23. Gent Test. Mol. Biomark..

[172] Ding Z.B., Shi Y.H., Zhou J., Qiu S.J., Xu Y., Dai Z., Shi G.M., Wang X.Y., Ke A.W., Wu B. (2008). Association of autophagy defect with a malignant phenotype and poor prognosis of hepatocellular carcinoma. Cancer Res..

[173] Amaravadi R. (2009). Autophagy can contribute to cell death when combining targeted therapy. Cancer Biol. Ther..

[174] Wei R., Cao G., Deng Z., Su J., Cai L. (2016). miR-140–5p attenuates chemotherapeutic drug-induced cell death by regulating autophagy through inositol 1,4,5-trisphosphate kinase 2 (IP3k2) in human osteosarcoma cells. Biosci. Rep..

[175] Lin B.C., Huang D., Yu C.Q., Mou Y., Liu Y.H., Zhang D.W., Shi F.J. (2016). MicroRNA-184 Modulates Doxorubicin Resistance in Osteosarcoma Cells by Targeting BCL2L1. Med. Sci. Monit..

[176] Lv C., Hao Y., Tu G. (2016). MicroRNA-21 promotes proliferation, invasion and suppresses apoptosis in human osteosarcoma line MG63 through PTEN/Akt pathway. Tumor Biol..

[177] Li J., Yen C., Liaw D., Podsypanina K., Bose S., Wang S.I., Puc J., Miliaresis C., Rodgers L., McCombie R. (1997). PTEN, a putative protein tyrosine phosphatase gene mutated in human brain, breast, and prostate cancer. Science.

[178] Chen J., Yan D., Wu W., Zhu J., Ye W., Shu Q. (2016). MicroRNA-130a promotes the metastasis and epithelial-mesenchymal transition of osteosarcoma by targeting PTEN. Oncol. Rep..

[179] Li B.L., Lu C., Lu W., Yang T.T., Qu X., Hong X., Wan X.P. (2013). miR-130b is an EMT-related microRNA that targets DICER1 for aggression in endometrial cancer. Med. Oncol..

[180] Colangelo T., Fucci A., Votino C., Sabatino L., Laudanna C., Binaschi M., Bigioni M., Maggi C.A., Parente D. (2013). MicroRNA-130b promotes tumor development and is associated with poor prognosis in colorectal cancer. Neoplasia.

[181] Li Z., Li Y., Wang N., Yang L., Zhao W., Zeng X. (2016). miR-130b targets NKD2 and regulates the Wnt signaling to promote proliferation and inhibit apoptosis in osteosarcoma cells. Biochem. Biophys. Res. Commun..

[182] Zhou Z., Wang Z., Wei H., Wu S., Wang X., Xiao J. (2016). Promotion of tumour proliferation, migration and invasion by miR-92b in targeting RECK in osteosarcoma. Clin. Sci. (Lond.).

[183] Takahashi C., Sheng Z., Horan T.P., Kitayama H., Maki M., Hitomi K., Kitaura Y., Takai S., Sasahara R.M., Horimoto A. (1998). Regulation of matrix metalloproteinase-9 and inhibition of tumor invasion by the membrane-anchored glycoprotein RECK. Proc. Natl. Acad. Sci. USA.

[184] Zutter M., Hockenbery D., Silverman G.A., Korsmeyer S.J. (1991). Immunolocalization of the Bcl-2 protein within hematopoietic neoplasms. Blood.

[185] Pietenpol J.A., Papadopoulos N., Markowits S., Wilson J.K., Kinzler K.W., Vogelstein B. (1994). Paradoxical inhibition of solid tumor cell growth by bcl2. Cancer Res..

[186] El-Emshaty H.M., Saad E.A., Toson E.A., Abdel Malak C.A., Gadelhak N.A. (2014). Apoptosis and cell proliferation: Correlation with BCL-2 and p53 oncoprotein expression in human hepatocellular carcinoma. Hepatogastroenterology.

[187] Ma C., Zhan C., Yuan H., Cui Y., Zhang Z. (2016). MicroRNA-603 functions as an oncogene by suppressing BRCC2 protein translation in osteosarcoma. Oncol. Rep..

[188] Fenger J.M., Roberts R.D., Iwenofu O.H., Bera M.D., Zhang X., Couto J.I., Modiano J.F., Kisseberth W.C., London C.A. (2016). MiR-9 is overexpressed in spontaneous canine osteosarcoma and promotes a metastatic phenotype including invasion and migration in osteoblasts and osteosarcoma cell lines. BMC Cancer.

[189] Paoloni M., Davis S., Lana S., Withrow S., Sangiorgi L., Picci P., Hewitt S., Triche T., Meltzer P., Khanna C. (2009). Canine tumor cross-species genomics uncovers targets linked to osteosarcoma progression. BMC Genomics.

[190] Guo L.M., Pu Y., Han Z., Liu T., Li Y.X., Liu M., Li X., Tang H. (2009). MicroRNA-9 inhibits ovarian cancer cell growth through regulation of NF-kappaB1. FEBS J..

[191] Zheng L., Qi T., Yang D., Qi M., Li D., Xiang X., Huang K., Tong Q. (2013). microRNA-9 suppresses the proliferation, invasion and metastasis of gastric cancer cells through targeting cyclin D1 and Ets1. PLoS ONE.

[192] Ni Z., Shang X.F., Wang Y.F., Sun Y.J., Fu D.J. (2016). Upregulated microRNA-301a in osteosarcoma promotes tumor progression by targeting CDC14A. Genet. Mol. Res..

[193] Su H., Jin X., Zhang X., Xue S., Deng X., Shen L., Fang Y., Xie C. (2014). Identification of microRNAs involved in the radioresistance of esophageal cancer cells. Cell Biol. Int..

[194] Xie H., Li L., Zhu G., Dang Q., Ma Z., He D., Chang L., Song W., Chang H.C., Krowleski J.J. (2015). Infiltrated pre-adipocytes increase prostate cancer metastasis via modulation of the miR-301a/androgen receptor (AR)/TGF-β1/Smad/MMP9 signals. Oncotarget.

[195] Zhou C., Tan W., Lv H., Gao F., Sun J. (2016). Hypoxia-inducible microRNA-488 regulates apoptosis by targeting Bim in osteosarcoma. Cell Oncol. (Dordr.).

[196] Li Y., Fan L., Liu S., Liu W., Zhang H., Zhou T., Wu D., Yang P., Shen L., Chen J. (2013). The promotion of bone regeneration through positive regulation of angiogenic-osteogenic coupling using microRNA-26a. Biomaterials.

[197] Fu X., Jin L., Wang X., Luo A., Hu J., Zheng X., Tsark W.M., Riggs A.D., Ku H.T., Huang W. (2013). MicroRNA-26a targets ten eleven translocation enzymes and is regulated during pancreatic cell differentiation. Proc. Natl. Acad. Sci. USA.

[198] Dey B.K., Gagan J., Yan Z., Dutta A. (2012). miR-26a is required for skeletal muscle differentiation and regeneration in mice. Genes Dev..

[199] Lu J., Song G., Tang Q., Yin J., Zou C., Zhao Z., Xie X., Xu H., Huang G., Wang J. (2016). MiR-26a inhibits stem cell-like phenotype and tumor growth of osteosarcoma by targeting Jagged1. Oncogene.

[200] Yin Z., Ding H., He E., Chen J., Li M. (2016). Up-regulation of microRNA-491–5p suppresses cell proliferation and promotes apoptosis by targeting FOXP4 in human osteosarcoma. Cell Prolif..

[201] Gong F., Ren P., Zhang Y., Jiang J., Zhang H. (2016). MicroRNAs-491–5p suppresses cell proliferation and invasion by inhibiting IGF2BP1 in non-small cell lung cancer. Am. J. Transl. Res..

[202] Guo R., Wang Y., Shi W.Y., Liu B., Hou S.Q., Liu L. (2012). MicroRNA miR-491–5p targeting both TP53 and Bcl-XL induces cell apoptosis in SW1990 pancreatic cancer cells through mitochondria mediated pathway. Molecules.

[203] Nakano H., Miyazawa T., Kinoshita K., Yamada Y., Yoshida T. (2010). Functional screening identifies a microRNA, miR-491 that induces apoptosis by targeting Bcl-X(L) in colorectal cancer cells. Int. J. Cancer.

[204] Zeng H., Chen Y., You W., Huang X.Q., Zhou C.Y., Li H. (2015). miR-491–5p functions as a tumor suppressor by targeting JMJD2B in ERα-positive breast cancer. FEBS Lett..

[205] Wang Y., Zhang S., Xu Y., Zhang Y., Guan H., Li X., Li Y., Wang Y. (2016). Upregulation of miR-192 inhibits cell growth and invasion and induces cell apoptosis by targeting TCF7 in human osteosarcoma. Tumor Biol..

[206] Li S., Li F., Niu R., Zhang H., Cui A., An W., Wang X. (2015). Mir-192 suppresses apoptosis and promotes proliferation in oesophageal squamous cell carcinoma by targeting. Bim. Int. J. Clin. Exp. Pathol..

[207] Geng L., Chaudhuri A., Talmon G., Wisecarver J.L., Are C., Brattain M., Wang J. (2014). MicroRNA-192 suppresses liver metastasis of colon cancer. Oncogene.

[208] Zhu Y., Wang W., Wang X. (2013). Roles of transcriptional factor 7 in production of inflammatory factors for lung diseases. J. Transl. Med..

[209] Chen W.Y., Liu S.Y., Chang Y.S., Yin J.J., Yeh H.L., Mouhieddine T.H., Hadadeh O., Abou-Kheir W., Liu Y.N. (2015). MicroRNA-34a regulates WNT/TCF7 signaling and inhibits bone metastasis in Ras-activated prostate cancer. Oncotarget.

[210] Zaidi N., Swinnen J.V., Smans K. (2012). ATP-citrate lyase: A key player in cancer metabolism. Cancer Res..

[211] Suagee J.K., Corl B.A., Crisman M.V., Wearn J.G., McCutcheon L.J., Geor R.J. (2010). De novo fatty acid synthesis and NADPH generation in equine adipose and liver tissue. Comp. Biochem. Physiol. B Biochem. Mol. Biol..

[212] Xin M., Qiao Z., Li J., Liu J., Song S., Zhao X., Miao P., Tang T., Wang L., Liu W. (2016). miR-22 inhibits tumor growth and metastasis by targeting ATP citrate lyase: Evidence in osteosarcoma, prostate cancer, cervical cancer and lung cancer. Oncotarget.

[213] Zhang C., Long F., Wan J., Hu Y., He H. (2016). MicroRNA-205 acts as a tumor suppressor in osteosarcoma via targeting RUNX2. Oncol Rep..

[214] Haxaire C., Haÿ E., Geoffroy V. (2016). Runx2 Controls Bone Resorption through the Down-Regulation of the Wnt Pathway in Osteoblasts. Am. J. Pathol..

[215] Chen H., Ghori-Javed F.Y., Rashid H., Serra R., Gutirrez S.E., Javed A. (2011). Chondrocyte-specific regulatory activity of Runx2 is essential for survival and skeletal development. Cells Tissues Organs..

[216] Yang G., Zhang P., Lv A., Liu Y., Wang G. (2016). MiR-205 functions as a tumor suppressor via targeting TGF-α in osteosarcoma. Exp. Mol. Pathol..

[217] Yao J., Qin L., Miao S., Wang X., Wu X. (2016). Overexpression of miR-506 suppresses proliferation and promotes apoptosis of osteosarcoma cells by targeting astrocyte elevated gene-1. Oncol. Lett..

[218] Ge L., Zheng B., Li M., Niu L., Li Z. (2016). MicroRNA-497 suppresses osteosarcoma tumor growth in vitro and in vivo. Oncol. Lett..

[219] Ruan W.D., Wang P., Feng S., Xue Y., Zhang B. (2016). MicroRNA-497 inhibits cell proliferation, migration, and invasion by targeting AMOT in human osteosarcoma cells. Oncol. Targets Ther..

[220] Holmgren L., Ambrosino E., Birot O., Tullus C., Veitonmäki N., Levchenko T., Carlson L.M., Musiani P., Iezzi M., Curcio C. (2006). A DNA vaccine targeting angiomotin inhibits angiogenesis and suppresses tumor growth. Proc. Natl. Acad. Sci. USA.

[221] Chen X., Chen X.G., Hu X., Song T., Ou X., Zhang C., Zhang W., Zhang C. (2016). MiR-34a and miR-203 Inhibit Survivin Expression to Control Cell Proliferation and Survival in Human Osteosarcoma Cells. J. Cancer.

[222] Pu Y., Zhao F., Cai W., Meng X., Li Y., Cai S. (2016). MiR-193a-3p and miR-193a-5p suppress the metastasis of human osteosarcoma cells by down-regulating Rab27B and SRR, respectively. Clin. Exp. Metastasis.

[223] Li W.H., Wu H.J., Li Y.X., Pan H.G., Meng T., Wang X. (2016). MicroRNA-143 promotes apoptosis of osteosarcoma cells by caspase-3 activation via targeting Bcl-2. Biomed. Pharmacother..

[224] Ning F.L., Wang F., Li M.L., Yu Z.S., Hao Y.Z., Chen S.S. (2014). MicroRNA-182 modulates chemosensitivity of human non-small cell lung cancer to cisplatin by targeting PDCD4. Diagn. Pathol..

[225] Liu R., Li J., Teng Z., Zhang Z., Xu Y. (2013). Overexpressed microRNA-182 promotes proliferation and invasion in prostate cancer PC-3 cells by down-regulating N-myc downstream regulated gene 1 (NDRG1). PLoS ONE.

[226] Bian D.L., Wang X.M., Huang K., Zhai Q.X., Yu G.B., Wu C.H. (2016). Expression and regulatory effects of microRNA-182 in osteosarcoma cells: A pilot study. Oncol. Lett..

[227] Zhang S., Zhao Y., Wang L. (2016). MicroRNA-198 inhibited tumorous behaviors of human osteosarcoma through directly targeting ROCK1. Biochem. Biophys. Res. Commun..

[228] Dong D., Gong Y., Zhang D., Bao H., Gu G. (2016). miR-874 suppresses the proliferation and metastasis of osteosarcoma by targeting E2F3. Tumour Biol..

[229] Song K., Liu N., Yang Y., Qiu X. (2016). Regulation of osteosarcoma cell invasion through osteopontin modification by miR-4262. Tumor Biol..

[230] Chen J., Zhu C., He Z., Geng M., Li G., Tao X., Zhang F. (2015). Association of OPN overexpression with tumor stage, differentiation, metastasis and tumor progression in human laryngeal squamous cell carcinoma. Int. J. Clin. Exp. Med..

[231] Li Y., Xie Y., Cui D., Ma Y., Sui L., Zhu C., Kong H., Kong Y. (2015). Osteopontin Promotes Invasion, Migration and Epithelial-Mesenchymal Transition of Human Endometrial Carcinoma Cell HEC-1A Through AKT and ERK1/2 Signaling. Cell Physiol. Biochem..

[232] Wang Z., He R., Xia H., Wei Y., Song W.U. (2016). MicroRNA-101 has a suppressive role in osteosarcoma cells through the targeting of c-FOS. Exp. Ther. Med..

[233] Liu M., Xu A., Yuan X., Zhang Q., Fang T., Wang W., Li C. (2015). Downregulation of microRNA-409-3p promotes aggressiveness and metastasis in colorectal cancer: An indication for personalized medicine. J. Transl. Med..

[234] Josson S., Gurutajan M., Hu P., Shao C., Chu G.Y., Zhau H.E., Liu C., Lao K., Lu C.L., Lu Y.T. (2014). miR-409–3p/-5p promotes tumorigenesis, epithelial-to-mesenchymal transition, and bone metastasis of human prostate cancer. Clin. Cancer Res..

[235] Wu S., Du X., Wu M., Du H., Shi X., Zhang T. (2016). MicroRNA-409–3p inhibits osteosarcoma cell migration and invasion by targeting catenin-δ1. Gene.

[236] Zhou Y., Han Y., Zhang Z., Shi Z., Zhou L., Liu X., Jia X. (2016). MicroRNA-124 upregulation inhibits proliferation and invasion of osteosarcoma cells by targeting sphingosine kinase 1. Hum. Cell..

[237] Wang L., Kang F.B., Sun N., Wang J., Chen W., Li D., Shan B.E. (2016). The tumor suppressor miR-124 inhibits cell proliferation and invasion by targeting B7-H3 in osteosarcoma. Tumor Biol..

[238] Chen G., Fang T., Huang Z., Qi Y., Du S., Di T., Lei Z., Zhang X., Yan W. (2016). MicroRNA-133a Inhibits Osteosarcoma Cells Proliferation and Invasion via Targeting IGF-1R. Cell Physiol. Biochem..

[239] Dong J., Liu Y., Liao W., Liu R., Shi P., Wang L. (2016). miRNA-223 is a potential diagnostic and prognostic marker for osteosarcoma. J. Bone Oncol..

[240] Li X., Liu X., Fang J., Li H., Chen J. (2015). microRNA-363 plays a tumor suppressive role in osteosarcoma by directly targeting MAP2K4. Int. J. Exp. Med..

